# Dual Coordination of Post Translational Modifications in Human Protein Networks

**DOI:** 10.1371/journal.pcbi.1002933

**Published:** 2013-03-07

**Authors:** Jonathan Woodsmith, Atanas Kamburov, Ulrich Stelzl

**Affiliations:** Otto-Warburg Laboratory, Max Planck Institute for Molecular Genetics (MPIMG), Berlin, Germany; University of Heidelberg, Germany

## Abstract

Post-translational modifications (PTMs) regulate protein activity, stability and interaction profiles and are critical for cellular functioning. Further regulation is gained through PTM interplay whereby modifications modulate the occurrence of other PTMs or act in combination. Integration of global acetylation, ubiquitination and tyrosine or serine/threonine phosphorylation datasets with protein interaction data identified hundreds of protein complexes that selectively accumulate each PTM, indicating coordinated targeting of specific molecular functions. A second layer of PTM coordination exists in these complexes, mediated by PTM integration (PTMi) spots. PTMi spots represent very dense modification patterns in disordered protein regions and showed an equally high mutation rate as functional protein domains in cancer, inferring equivocal importance for cellular functioning. Systematic PTMi spot identification highlighted more than 300 candidate proteins for combinatorial PTM regulation. This study reveals two global PTM coordination mechanisms and emphasizes dataset integration as requisite in proteomic PTM studies to better predict modification impact on cellular signaling.

## Author Summary

Normal cellular functioning is maintained by a vast array of macro-molecular machines that control both core and specialised molecular tasks. These machines are in large part multi-subunit protein complexes that undergo regulation at multiple levels, from expression of requisite components to a vast array of post translational modifications (PTMs). PTMs such as phosphorylation, ubiquitination and acetylation currently number up to more than 100,000 in the human proteome yet how, or if, they coordinate remains poorly understood. Here we show two mechanisms of systematic modification coordination that likely combine to provide finer control of protein complex function. Firstly, individual modifications selectively target protein complexes to execute specific molecular functions. Secondly, highly modified subunits of these complexes further accumulate multiple distinct modifications and contain regions of dense modification patterns, termed PTM integration (PTMi) spots. Through multiple PTM inputs, PTMi spots represent key regions for integrating multiple signals within these complexes, allowing finer regulation of protein function. Here we highlight the large extent of coordinated PTM regulation of protein complexes, and hence cellular function. Systematic dataset integration revealed biological insight into PTM mediated cellular regulatory mechanisms and further provides a resource for future hypothesis-driven studies.

## Introduction

Normal cellular functioning requires a broad range of rapid responses to both internal and external cues that are largely mediated through multiple proteins acting in coordination to undertake specific molecular tasks. These rapid responses are predominantly mediated through alteration of protein binding partner preferences, stability and activity via regulation by a vast array of post-translational modifications (PTMs).

Human PTMs are known to number greater than 400 [1] and range from small chemical modifications of amino acid side chains such as acetylation [2] and phosphorylation [3] to the addition of the large peptide chains of the ubiquitin and ubiquitin-like families through isopeptide bonds [4]. PTM regulation is achieved by a large number of components encoded by 5–10% of the protein coding genome, each controlled by distinct regulatory systems that vary in both size and mechanism of modification. For example, reversible protein phosphorylation is controlled through the direct action of >500 kinases [5] and >150 phosphatases. Under 100 deubiquitinating enzymes mediate the direct removal of distinct forms of ubiquitin [6] while in contrast to direct kinase action, >600 components mediate target protein modification in a more combinatorially complex enzymatic cascade [7], [8]. The critical requisite for normal PTM functioning can be observed as many regulatory proteins are annotated in disease pathogenesis and as such are the targets of current drugs or in ongoing clinical trials [9]–[11]. Given their number and diversity, it is unsurprising that these modifications cover a huge range of molecular functions.

A further layer of PTM complexity is generated through interplay between modifications on the same protein. This interplay, or crosstalk, can either modulate the occurrence of distinct PTMs at the same or spatially separated sites, or act in concert to generate combinatorial outputs. Directed studies have provided several functional paradigms for PTM interplay in a range of cellular processes from modification of histone tails in epigenetic control [12], gene transcription by RNApol II [13], cell fate orchestration by TP53 [14] to dynamic control of signaling through the EGFR [15].

Systematic analysis and identification of novel candidates for combinatorial PTM regulation has been predicated by data paucity for multiple signaling PTMs. Identification of phosphorylation sites on both tyrosine (pY) or serine/threonine (pS/T) residues has been aided by the generation of biochemical affinity reagents [16], [17] or pan-specific pY antibodies [18], [19] allowing systematic enrichment of these modifications from total cell lysates. However, global identification of other PTMs had lagged behind. Recently multiple high throughput proteomic studies have revealed acetylation (Ac) and ubiquitination sites (or Neddylation, for simplicity further referred to as solely ubiquitination (Ub)) spanning a large proportion of the proteome [20]–[24]. Each of the above studies used novel modification specific antibodies to enrich cellular lysates to be able to identify targets sites systematically, aiding much broader coverage of modified proteins than was previously possible. Across all four modifications (pS/T, pY, Ac and Ub), these systematic and small scale studies have identified over 100,000 target sites distributed across >12,000 unique human proteins. While these four PTMs are well characterized to be mediated and recognized by distinct regulatory systems (for a review see [25]) their interplay in control over multiple cellular functions is poorly studied.

Two recent studies utilised evolutionary conservation of PTMs to drive systematic investigation into modification co-evolution [26] and functionality [27], focusing on predicting characteristics of PTMs within individual proteins in isolation. However, all cellular processes are governed by protein interactions [28], [29], a feature which has not been addressed in these two studies. Previously it has been shown that phospho-proteins in yeast are more likely to interact than expected by random chance [30], a characteristic also shown by modified prokaryotic proteins [31]. Also, phosphorylation is coordinated in protein interactions networks in response to epidermal growth factor [32]. Furthermore, acetylation has been shown to accumulate over protein complexes in human cultured cells [24]. Therefore we took a protein network driven approach and systematically characterized four globally measured PTMs through integration of >100,000 PTMs with protein complex data, highlighting emergent features of PTM signaling in humans: Protein complexes accumulate specific PTMs through selective interaction profiles and these complexes are enriched for subunits that are modified by multiple PTMs. Furthermore, while this PTM signal is generally spatially distributed, highly dense PTM integration (PTMi) spots are preferentially found within these complexes, showing both single- and multi-PTM signal integration. This infers these complexes are regulated through combinatorial PTM inputs in two distinct layers; accumulation of specific PTMs through selective binding profiles and via PTMi spot containing proteins.

## Results

### Identification of PTM enriched protein complexes

All PTM data was collated into a single dataset of 100,391 modifications, distributed across 12,127 proteins (**Table 1**, **Dataset S1**, for details of collation and dataset characteristics see Materials and Methods). This dataset provides a resource for further investigation into the selectivity and functionality of four different PTMs on a global scale. No protein in the cell functions in isolation, rather each interacts with or is embedded within multi-subunit protein complexes to carry out requisite molecular tasks. Therefore we integrated this PTM resource with human protein complex data from ConsensusPathDB [33] to ascertain how multiple modifications are coordinated within a functional human protein interaction network. Taking forward only protein complexes of three or more unique subunits this resulted in a dataset of 4462 complexes constituted by 4143 proteins (**Dataset S2**, for details of collation and dataset characteristics, see Materials and Methods).

**Table 1:** Post translational modification datasets.

	All Modified Components		Complex Components	
	IDs	Total PTMs	IDs	Total PTMs
**Ac**	3009	6682	1241	3370
**pS/T**	8859	56251	2863	24794
**pY**	5666	13241	1956	5865
**Ub**	6244	24217	2265	11677
**Totals**	**12127**	**100391**	**4143**	**45706**

Number of unique proteins and the number of modifications across the proteome or in defined human complexes.

**Figure 1:**
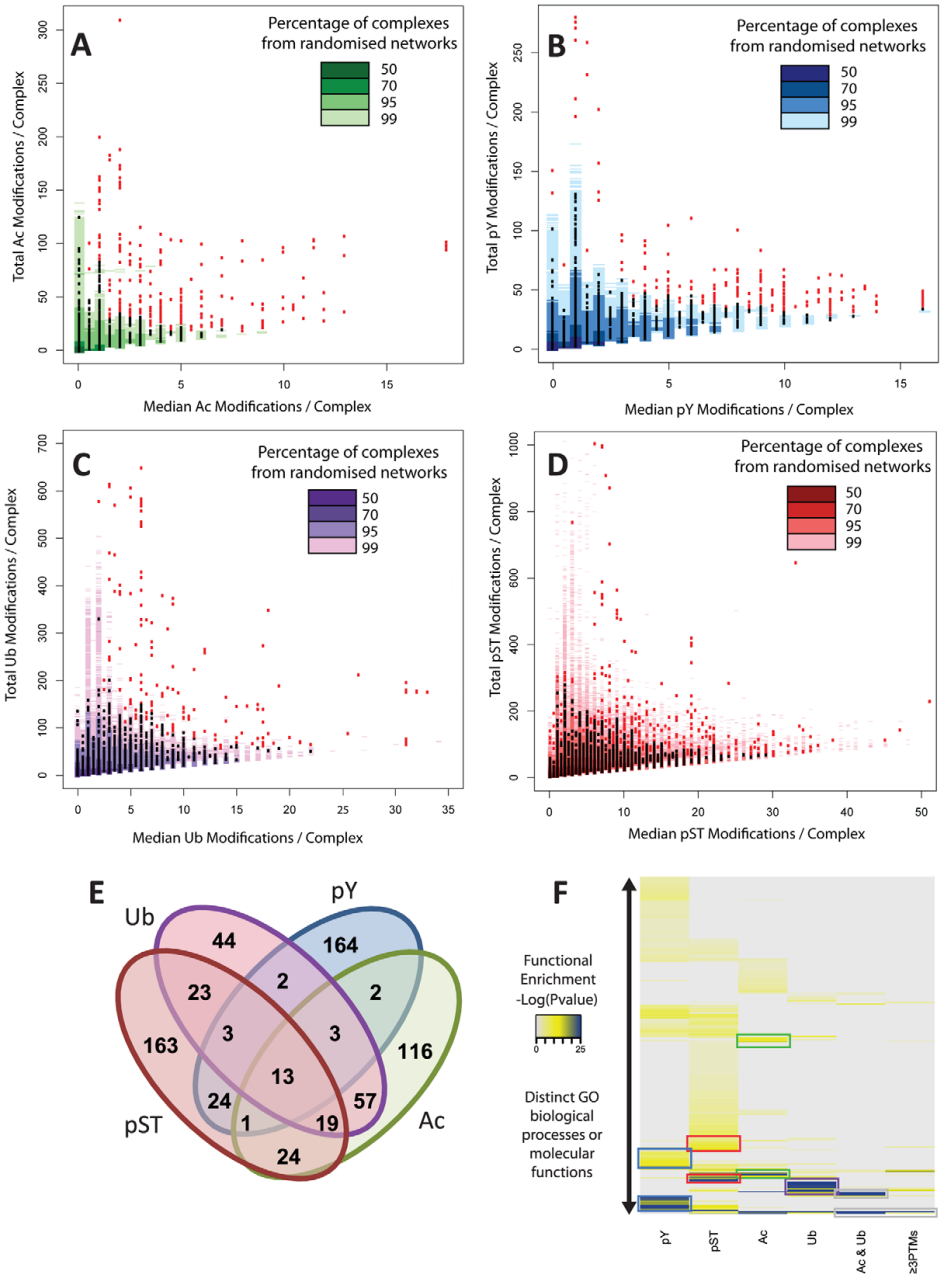
Selectivity of post-translational modification on human protein complexes. Median modification level of protein complexes plotted against total number of modifications for each PTM, with each data point representing a unique complex for (**A**) acetylation, (**B**) tyrosine phosphorylation, (**C**) ubiquitination and (**D**) serine/threonine phosphorylation. Data points may overlay preventing visualisation of each unique complex. The density data from 100 random datasets is overlaid on each plot with graded colours representing percentages of total randomized data. Complexes that are highly unlikely to be generated through random dataset generation are selected at confidence cut-offs of 99% for Ac, pY and Ub and 95% for pS/T and highlighted in red. (**E**) Overlap analysis for selected, highly modified complexes from 1A–D. (**F**) GO analysis highlighting coordinated differential molecular function control by each group of complexes selected in the above analysis. Ac & Ub represents the 57 complexes that are enriched for both these PTMs, ≥3PTMs represents the 39 complexes enriched in at least 3 PTMs.

In analogy to individual proteins that can be functionally classified by overall modification status [34]–[36], we reasoned protein complexes could likewise follow similar regulation. We therefore investigated the prevalence of highly modified complexes for each modification that could represent targets for concerted PTM regulation. Protein complexes are distributed over a vast range of sizes, to distinguish between complexes that are large and thus more likely to accumulate modifications and smaller complexes that may contain a high proportion of modified subunits we plotted the total modification level of any given complex against its median (**Figures 1** **and S1**). The majority of protein complexes were poorly modified for any given PTM and there were only 2 complexes that showed both a high total number and high average level of modification, with one NFAT5 transcriptional regulatory complexes in the ubiquitination dataset and an SRRM1 containing, phospho-serine/threonine enriched, regulatory splicing complex (Red circles, **Figures S1C–D**). Interestingly however, two distinct groups of protein complexes can be observed that were separated from the majority distribution (**Figure S1**). For all four modifications we found complexes that tend towards a large total modification but low median, and those that had fewer modifications but a higher median (**Figures 1A–D**). For each modification the two groups of highly modified complexes can be generally characterized as huge complexes with many modified subunits or smaller complexes with high modification levels of its components (**Figure S2**).

To ascertain whether these highly modified complexes are likely to occur through the random accumulation of PTMs across protein complexes, the datasets were compared to a null model obtained through PTM annotation permutation. We sub-divided the proteins into 16 distinct bins, controlling for both protein size and frequency in the dataset, and then permuted the PTM annotations for all proteins within a bin (**Figure S3**). The enriched groups of complexes for each PTM showed a high modification state distinct from 99% of data generated through 100 dataset randomisations (**Figures 1A–D**, red data points on graphs, 95% cut-off taken for pS/T). Despite completely distinct regulatory mechanisms governing each of acetylation, tyrosine phosphorylation, ubiquitination and serine/threonine phosphorylation, these groups of highly modified complexes show similarities in numbers with 160–270 complexes separated from the majority distribution.

Next we asked if these complexes are highly modified by several different modifications through examining the overlap between the four sets of enriched complexes. The complexes highly modified by one type of PTM were largely distinct (**Figure 1E**), with only acetylation and ubiquitination showing a larger degree of overlap. Complexes targeted by acetylation had been noted previously [24], however this is the first evidence to suggest that Ac and Ub not only target the same amino acid but coordinate over entire complexes. 39 of the 659 complexes were present in 3 or more of the enriched datasets, representing target points for regulation through multiple signals within the cell.

To examine the functions of the targeted complexes we undertook a Gene Ontology analysis using the ConsensusPathDB over-representation tool [33]. Acetylation enriched complexes were involved in sequence specific DNA binding, transcription coactivator activity and metabolic processes (Green boxes, **Figure 1F**, full size image **Figure S4**). Phospho-serine and phospho–threonine complexes were involved in multiple mitotic stages, metabolism and cellular spatial organisation (Red boxes, **Figure 1F**). Phosphorylation on tyrosine residues showed the most distinct number of highly modified complexes with GO terms relating to response to extra-cellular stimulation, cell migration and immune cell functions (Blue boxes, **Figure 1F**). Complexes enriched for ubiquitination were involved in S phase and interphase cell cycle control, catabolic processes and the DNA damage response (Purple box, **Figure 1F**). The highlighted complexes support ubiquitin as a multifunctional modification, with stability control (cell cycle) and non-degradative functions (DNA damage) both enriched. Given that a large proportion of ubiquitin chains in the cell are not classical Lys-48 linked proteasomal signals [22], we hypothesize that these complexes may be targeted with multi-functional/distinct ubiquitin signals. Complexes enriched for multiple PTMs were involved in mRNA regulation, predominantly through modification of spliceosomal and ribosomal sub-complexes (Grey boxes, **Figure 1F**). Therefore each of these subgroups of specifically modified protein complexes mediates distinct cellular processes. These results are in excellent agreement with the current knowledge base of functional regulation for each modification supporting our approach to PTM complex modification identification.

### Signal integration on highly modified complexes

**Figure 2:**
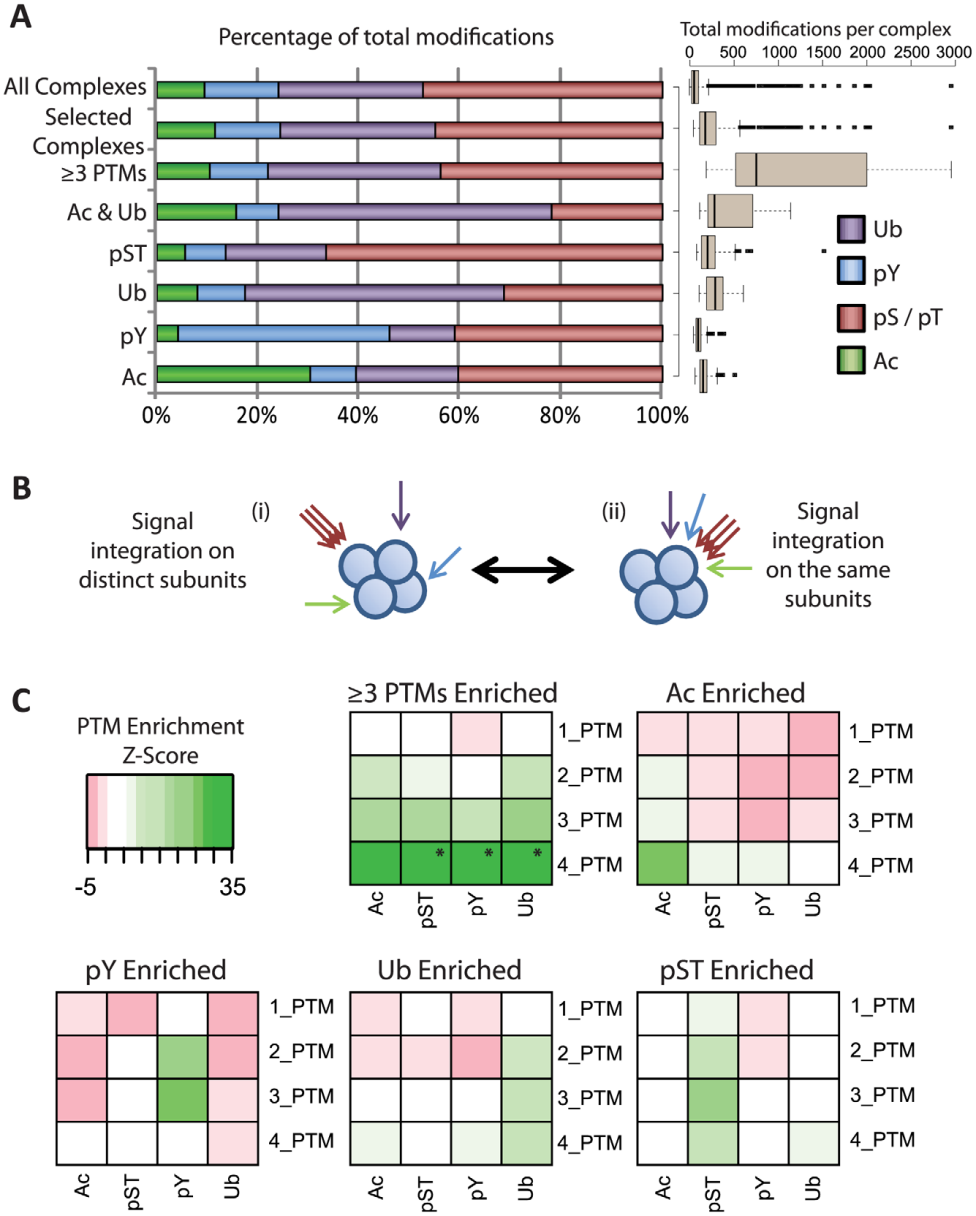
Signal integration on highly modified protein complexes. (**A**) Percentage of total modifications across each specific group of complexes in comparison to all complexes in the dataset and all of the enriched complexes combined (Selected Complexes). Box plot represents the total number of modifications present across all complexes for each enriched group. (**B**) Schematic representations of potential PTM integration. Putative signal integration through distinct singly-modified subunits (i) is compared to more complex combinatorial signaling through multiply-modified subunits (ii). (**C**) Enrichment analysis for each subgroup of nodes. Each square represents the Z-score for an increase or decrease in signal compared to random samples from the entire complex dataset. * Represents Z-scores >35, not reflected by the colour code of the heatmap.

Having observed this coordination of specific PTMs on a functional protein complex level we next sought to characterize these complexes with respect to the levels of other types of modification. Interestingly, while these complexes are enriched for one PTM they are also modified by the other three PTMs, however the distribution of total modifications in these complexes is shifted towards a larger percentage of the enriched modification (**Figure 2A**). Exemplarily, complexes enriched for acetylation contained a greater number of phosphorylated serine and threonine residues than acetylated lysines, due to the large number of phosphorylated residues in the dataset. The 39 complexes that were enriched for 3 or more PTMs (≥3PTMs) contained the same proportion of modifications as all complexes in the dataset but were very heavily modified with an average of over 500 modifications per complex (**Figure 2A**). To investigate the nature of this signal integration across protein complexes we therefore asked whether the enriched modification signal is carried by distinct subunits modified with one type of PTM each (**Figure 2B(i)**), or whether multiple PTMs are directed onto single proteins in these complexes (**Figure 2B(ii)**). For each group of complexes, all subunits were initially divided into sub groups based on their number of distinct modifications (1–4 PTMs). The total modification across all proteins within a sub group was then determined for each modification. This was compared to the signal carried by random samples of the same number of complexes from the entire dataset. Thus we could observe the origin of this modification enrichment, and see whether it preferentially stemmed from multiply modified components or from components with only one type of modification (**Figures S5, S6, S7, S8, S9**). For example while there were 28 proteins only modified by pS/T in the acetylation enriched complexes, the total number of modified residues (133, **Figure S5**) is less than expected from random samples of the whole dataset (**Figure 2C**, Ac: 1_PTM row, pST col, pink). On the other hand we observed a strong Ac signal for the proteins that also carried the other three modifications (**Figure 2C**, Ac: 4_PTM row, Ac col, green). Furthermore, complexes enriched for ≥3PTMs carried much more signal than random samples from the entire dataset on multiply modified proteins, *i.e.* on proteins modified by 3 or 4 different PTMs (**Figure 2C**). **Figure 2C** further highlights that for each group of enriched complexes the PTM enrichment was found in the respective column and that proteins with ≥2 distinct PTMs carried the signal, supporting the model where PTM integration takes place on individual subunits (**Figure 2B(ii)**). This suggests that while these complexes are identified as highly modified for a specific modification, the proteins that contribute most to the elevated modification levels of the complexes are controlled by complex PTM regulation.

Interestingly, in contrast to the other modifications, pY enriched complexes contained a much larger signal enrichment on proteins modified only by 2–3 PTMs than by those modified by 4 PTMs. In agreement with the previous complex level analysis (**Figures 1E and F**), phospho-tyrosine modifications were in large part exclusive to both acetylation and ubiquitination. 83% of total modification across these proteins was phosphorylation with 96% (276/289) of proteins modified by 2 PTMs being solely phosphorylated. This suggests that complexes that are enriched for pY signaling undergo regulation predominantly through phosphorylation.

### Dense PTM regions on complex components

**Figure 3:**
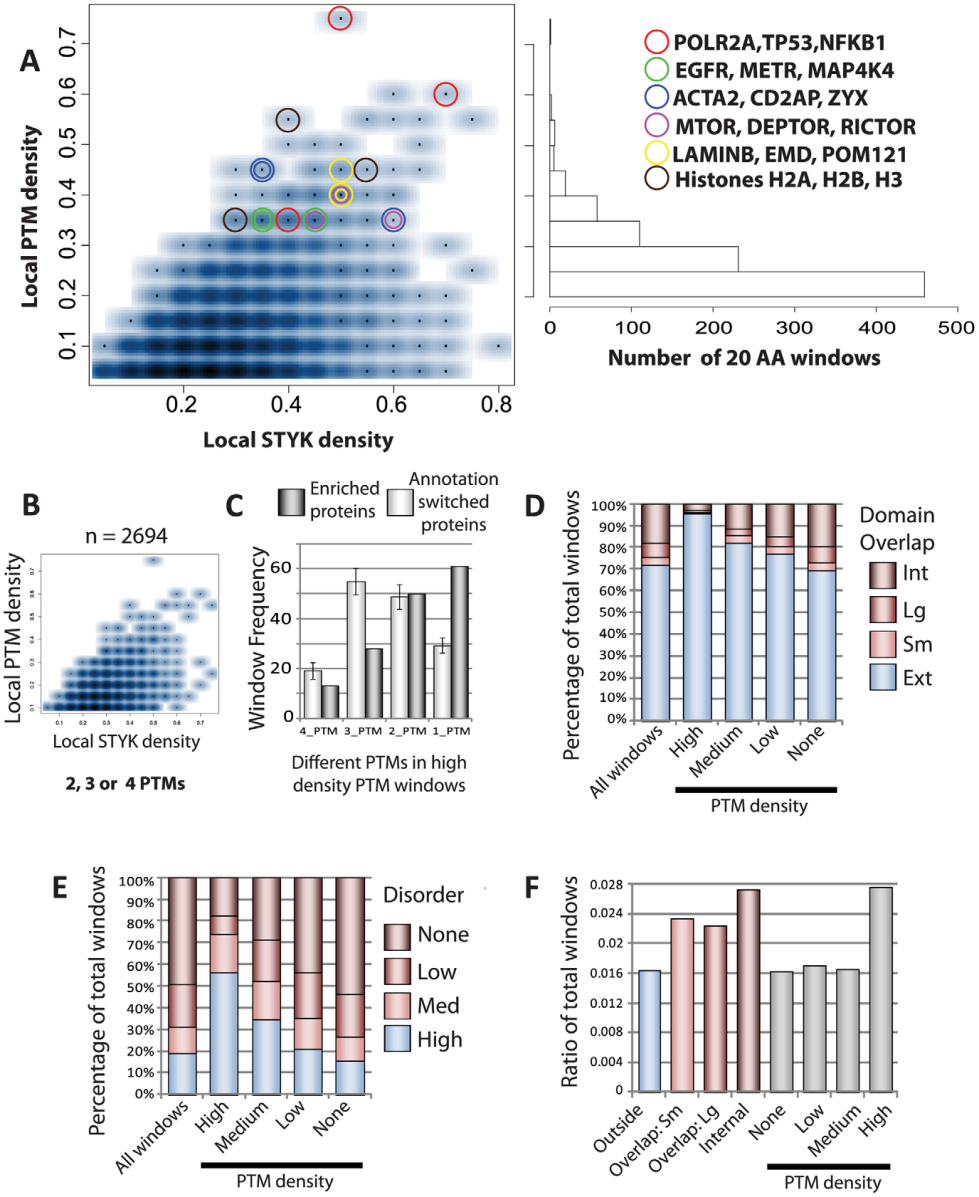
PTMi spot identification and characterisation. (**A**) 2D density plot of local STYK AA density windows plotted against local PTM density with a histogram showing the number of local peaks. The 500 most outlying data points are plotted as points on the density plot, however with substantial overlay, preventing visualization of all data point. Some PTM regions contain a higher PTM density than modifiable residues, highlighting regions which are key candidates for direct ubiquitination:acetylation competition on the same lysine residues. Specific proteins with densely modified regions are indicated through coloured circles. (**B**) 2D density plot of the number of 20 AA windows containing more than 1 PTM. (**C**) The number of high density windows that contain multiple PTMs compared to 100 random annotation permutation simulations. (**D**) Overlap analysis between individual PTM 20AA windows and annotated protein domains. Int: 20AA window internal to a protein domain, Lg: Large overlap with a proteins domain (>10AAs), Sm: Small overlap with a protein domain, Ext: 20AA window external to an annotated protein domain. (**E**) Overlap analysis between individual PTM 20AA windows and predicted protein disorder. High: Every amino acid is predicted to be disordered, Med: 11–19 AAs in a window are predicted to be disordered, Low: 1–10 AAs in a window are predicted to be disordered. (**F**) Frequency of 20AA windows across a protein sequence that are mutated in cancerous cells, sorted based on their protein domain annotation (coloured bars). The frequency of 20AA windows outside of annotated protein domains that are mutated in cancerous cells, sorted based on their PTM density (Grey bars).

Given these complexes contained proteins highly modified by more than one type of PTM we then further investigated the PTM signal integration potential of those complex subunits that were modified with ≥2 distinct types of PTMs. While PTM interplay can likely occur between distal modifications on the same protein sequence we reasoned that clusters of modifications would more directly promote combinatorial PTM regulation, such as those extensively studied on histone H3/H4 N-terminal tails (for a review see [12]). To identify regions of PTM clusters, the local PTM density was calculated scanning with overlapping sequence windows of 20 amino acids (AAs) across each protein. Non-overlapping, local peaks of PTM density were then calculated for each protein to give a total of 10,004 20AA peaks in 1361 unique proteins. The density of lysine (K), tyrosine (Y), serine (S) and threonine (T) residues was also calculated to correlate PTM density values with the proportion of modifiable residues present in each window. **Figure 3A** highlights that while the majority of 20AA windows contained low local PTM density indicating spatial separation of PTMs, hundreds of high density regions were present throughout proteins in PTM enriched complexes. 207 local peaks spread across 147 proteins contained a density of more than one modification for every 3 amino acids over a stretch of 20 amino acids, highlighting the prevalence of defined sequence space that have a high potential to integrate multiple PTMs and produce combinatorial outputs.

In support of the hypothesis that proteins with these regions represent candidates for complex PTM regulation, this scoring metric highlighted canonical examples of regulation through PTM interplay. TP53, POLR2A, multiple histones and RTKs (EGFR) were all present among proteins with regions of high PTM density (PTM density >0.3; **Figure 3A**). The frequency of novel candidate proteins identified alongside the canonical examples of PTM interplay suggests it is not limited to these well studied examples but rather plays a broad role in cellular functioning. Candidate proteins included many well characterized proteins involved in transcription (NFKB1, **Figure 3A**), cellular signaling (MET, MAP4K4), cytoskeletal dynamics (ACTA2, CD2AP, ZYX), mTor signaling (MTOR, DEPTOR, RICTOR) and nuclear membrane proteins (LAMINB, EMD, POM121). These dense PTM peaks contained examples of both single-PTM (one type of modification) and multiple-PTM (different types of modification) signal integration, with regions that contained multiple-PTMs showing an overall similar distribution to all modified regions (**Figure 3B**) and representing 57% of highly dense regions (**Figure S10A**).

The highly dense PTM regions showed a similar contribution of each PTM to that present in all PTM modified windows (**Figure S10B**). We therefore sought to investigate how stringently controlled multiple-PTM integration is within these regions using a permutation analysis. We randomised the PTMs annotated at specific sites across a protein sequence, controlling for both PTM density and type and then calculated the number of different PTMs present in 20AA windows across all proteins in 100 permutations. As can be seen in **Figure 3C**, there were fewer highly dense peaks (PTM density >0.3) that contained multiple-PTMs and a greater number that contained single-PTM integration alone than expected from random PTM annotation permutation. This result again suggests stringent control of multiple signal integration and shows that multiple-PTM dense regions do not occur through random accumulation of PTMs across proteins. As well exemplified for POLR2A or TP53 (red circles, **Figure 3A**), multiple-PTMs densely integrated in specific regions are therefore likely key to the regulation and function of those proteins.

We hypothesized that in analogy to functional, sequence defined domain architectures, whereby single proteins combine multiple domains in a modular fashion for increased functionality, spatially separated peaks of high PTM density in individual protein sequences may have analogous properties. We therefore analyzed these local PTM peaks in the context of the whole protein sequence. In general, PTM peaks were distributed unevenly across proteins with most containing very few high density peaks (**Figure S10C**), showing diversity in both density and length (**Dataset S3**). We therefore sought to operationally define and characterize high density regions with respect to overall protein architecture. Sequential 20AA windows across the entire protein sequence were utilised to ascertain the overlap between PTM dense regions and annotated protein domains (**Dataset S3**). Highly dense PTM windows were predominantly located outside of classically annotated functional domains (blue bars, **Figure 3D**), with lower PTM density windows showing a higher percentage of windows that either overlap, or are internal to, annotated domains (**Figure 3D**). While S/T phosphorylation has been previously shown to accumulate in disordered regions of proteins across multiple species [37]–[39], acetylation has been conversely shown to accumulate in more ordered regions of protein structure [24]. Therefore we investigated how the multiple PTMs analysed here were distributed across regions of predicted protein disorder outside of sequence defined protein domains. ∼83% of 20AA windows with high PTM density contained some disordered protein sequence with ∼56% being completely disordered (**Figure 3E**). The fraction of 20AA windows associated with disordered sequence decreased with PTM density highlighting that regions of dense modifications preferentially accumulate in disordered regions outside of annotated protein domains.

Mutations in functional protein regions can lead to oncogenic transformation of human cells and would therefore be expected to occur in high frequency in domains when querying systematic sequencing datasets. Following this line of thought we sought to compare dense PTM regions and annotated domains and integrated 182,581 mutations from the COSMIC cancer database [40] with the local PTM density data. The frequency of mutations in cancer cells will be generally higher in functional regions but can vary by an order of magnitude within 20AA windows (**Dataset S3**). Therefore the dataset was binarised, regions with 0 or 1 mutation were treated as background and windows with 2 or more mutations were taken forward (11.5% of all mutations). The fraction of mutated 20AA windows in annotated protein domains was higher in comparison to windows covering either fringe or external regions of domains (coloured bars, **Figure 3F**). As a general trend this infers functional relevance to these annotated domains over other protein coding regions in normal cellular functioning. However, as described above the majority of dense PTM regions lie in regions external to these domains (**Figure 3D**). We therefore analysed the mutation window frequency outside of annotated domains, further sub-dividing windows into bins of increasing PTM density (grey bars, **Figure 3F**). A strikingly high ratio of mutated windows was observed in the high density PTM regions. The value was equal to annotated functional domains, suggesting an equivocal importance in normal cellular function. Importantly, this trend was also observed when using either a higher cut-off for dataset binarisation or only complex components that are annotated as either tumor suppressors or oncogenes **(Figure S11)**.

Taken together, identification of canonical examples of PTM interplay, stringent control of multiple-PTM integration, spatial separation from annotated protein domains into regions of protein disorder and the high cancer mutation frequency all support the notion that the highly dense regions of PTMs bear functionally relevant features and can thus be characterized as PTM integration (PTMi) spots. Recently, Beltrao et al. [27] had used the term “hot spot” to describe modified residues within protein domains that likely represent functional PTMs. “PTMi spots” here represent clusters of modifications (both single- and multiple-type modifications) in regions of short sequence space to integrate multiple PTMs. Using the correlations observed we defined, at a PTM density cut-off of >0.3, 201 PTMi spots distributed across 147 proteins between 20 and 90 AAs in length (**Dataset S4**). The proteins showed diverse PTM density distributions with some dominated by a single-PTMi spot, such as the highly studied C-terminal tail of POLR2A crucial for transcriptional initiation and elongation, while others showed two or more (**Dataset S4**). PTMi spots provide regulatory focal points within these proteins as sites of single- and multiple-PTM integration and they preferentially accumulate in the highly modified complexes identified here (**Figure S12**), providing a second layer of PTM coordination within protein complexes.

### Proteome wide PTM integration spot (PTMi spot) characterisation

Interestingly, while combining the PTM data with known protein complexes provided insight into two distinct layers of PTM coordination within protein networks, PTMi spot characteristics were also observed throughout the entire PTM dataset (405 PTMi spots across 323 proteins, **Figure S13** and **Dataset S4**), suggesting that this mechanism of PTM coordination exists proteome wide.

Therefore we sought to further characterize the PTMi spots independently of the protein complex data and initially investigated the influence of local disorder and STYK ratios on PTMi spots proteome wide in more detail. While the fraction of disordered windows increased as the STYK ratio increased, the total number of disordered 20AA windows actually decreased (**Figure S14A**). However, the percentage of windows that were modified across STYK density varied only slightly between 20–27% (**Figure S14B**). While the majority of these windows contained few modified residues, the PTM distribution shifted to more dense modification patterns with increasing STYK density (**Figure S14B**). Therefore, as expected, the number of windows containing a PTM density of >0.3 initially increased, with higher STYK densities allowing more dense modification patterns. However, the total number of available windows becomes limiting at higher STYK densities, leading to the observation of a peak of highly modified windows between 0.4–0.5 local STYK density (**Figure S14C**). Disordered windows were favored at high modification states at the STYK density of 0.4–0.5, showing a 2–3 fold increase (**Figure S14C**). We therefore observed that the number of windows at a given STYK density guides the presence of highly modified protein sequence, and that within the optimal STYK density, disordered regions create much more favorable conditions, e.g. the structural flexibility, for multiple modifying enzymes to act.

**Figure 4:**
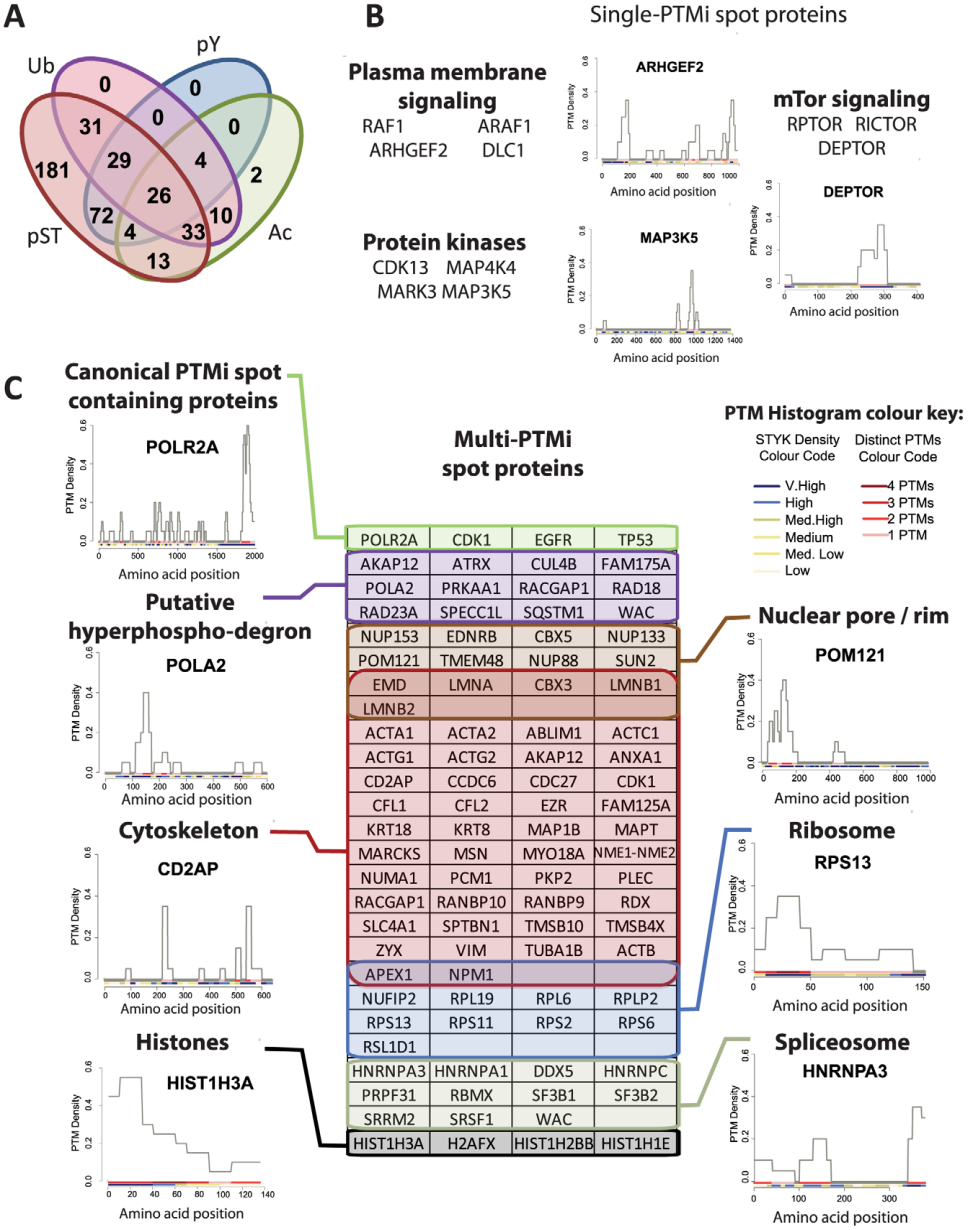
PTMi spot containing proteins. (**A**) Breakdown of the PTMs present in each of the 405 PTMi spots identified here. (**B**) Examples of single phospho-PTMi spot containing proteins with histograms of the local PTM density across the protein sequence. Colour codes beneath each density histogram represent the number of distinct PTMs (red scale) and the local STKY density (yellow∶blue scale, see legend [is in C]). (**C**) Multi-signal PTMi spot proteins. Examples of proteins annotated either as canonical PTMi spot containing protein, a protein that integrates both ubiquitin and multiple phosphorylations in a PTMi spot (putative degron), or to one of five key regulatory cellular modules. Representative examples of PTM density distributions of a protein in each sub-group is linked to the panel, a full list of PTMi spot containing proteins is found in Dataset S4.

Disordered protein regions often contain common short (3–10 residues) linear motifs (SLIMs) that can provide mechanistic insight into protein function. We parsed the PTMi spot sequences using the SLIMfinder tool [41] to identify over-represented SLIMs associated with these regions. 17 of the 20 over-represented SLIMs could be assigned to proline directed kinase motifs (**Dataset S4**), with the only characterized SLIM of the remaining three representing a PKB motif. The enrichment for target sites for serine/threonine kinases here is unsurprising given that the majority of single-PTMi spots were phosphorylated on serine or threonine residues (**Figure 4A**). Each single-PTMi spot contains a minimum of 7 pS/T residues, or 128 (2^7^) possible modification states, thus it is likely that phosphorylation either proceeds in a sequential manner or not all states have distinct functional outcomes [42]. Regions of bulk pS/T have been previously observed in disordered regions of human proteins [37]–[39] and have the potential to mediate their functionality through defining local electrostatic patterns. Proximal multisite phosphorylation has been modeled to function as an ultrasensitive switch [43] regulating nuclear import, membrane localization, stability and DNA binding (for a review see [42]). For example, the SRR1 motif of NFATC2, here highlighted as a single-PTMi spot, was previously shown to be hyperphosphorylated in the cytoplasm. Upon dephosphorylation of the SRR1 motif NFATC2 undergoes a conformational change and is transported to the nucleus [44]. Also, bulk charge in defined regions has been shown previously to be important for the localization the yeast proteins Ste5 [34], whereby multiple proximal phosphorylations preclude binding to the membrane and attenuate signaling. We therefore hypothesize that many of the >180 single-PTMi spots identified here (**Dataset S4**) could regulate localization of target proteins. For example, in analogy to Ste5, the ARAF, RAF1, ARHGEF2 and DLC1 proteins are involved in signaling from the outer cell membrane (**Figure 4B**). Furthermore, kinases themselves and mTor pathway components have PTM landscapes dominated by single-PTMi spots that could likewise regulate their localization (**Figure 4B**).

The potential for single-PTMi spots to regulate protein localization then led us to investigate the potential for multi-PTMi spots to perform likewise functions. A sub-class of nuclear localization signals (NLSs) have been reported in disordered regions of protein sequences and require a R/H/KX_(2–5)_PY motif downstream of either a hydrophobic or basic stretch of amino acids [45]. Here we report four putative PY-NLSs in HNRNPA3, SRRM2, TRA2B, and BCLAF1 that integrate multiple signals and thus could control protein localization (**Figure S15**). Phosphorylation of serine residues in the canonical HNRNPA1 PY-NLS has been previously reported to reduce binding affinity of karyopherins/importins and was also observed in each putative PY-NLS here. Interestingly, the tyrosines in the strictly conserved PY motifs were phosphorylated in the four candidate proteins. These tyrosines have been shown to be crucial for importin binding and make several hydrophobic interactions with karyopherin β [45] (**Figure S15**), suggesting distinct signals may integrate within these PTMi spots to regulate protein localization.

The yeast protein Sic1 provides a functional paradigm for phosphorylation and ubiquitination integration that can be further analyzed here. During the S phase of the cell cycle, lysine residues are ubiquitinated in response to proline directed hyperphosphorylation of adjacent serine residues, ultimately leading to Sic1 proteosomal degradation [36], [46]. Here we can identify 47 multi-PTMi spots that contained ≥4 pS/T residues, at least one of which is within a proline directed kinase motif, and also contained ≥1 annotated ubiquitination sites (a subset of these displayed in Figure 4C as “putative hyperphospho-degron”, for a full list see **Dataset S4**). This suggests that these PTMi spots may be hyperphosphorylation switches that mediate stability of many human protein targets.

Besides localization and stability control, the 240 multi-PTMi spots identified proteome wide include the few well characterized examples of multiple PTM interplay in human cells. Functional paradigms provided by intensively studied proteins such as TP53, RNApol II subunit POLR2A and core histones demonstrates the regulatory potential of combinatorial PTMs [47] (**Figure 4C**). Histones in particular represent intensively studied examples of multiple signal integration used to provide docking for multiple epigenetic regulators and as signaling scaffolds in context dependent gene transcription control (for a review see [12]). Analogously, an array of cytoskeletal proteins/regulators were annotated as containing multi-PTMi spots, supporting the notion that structures at the plasma membrane likewise represent dynamic signaling scaffolds for multiple context dependent signal integration (**Figure 4C**, for a review see [48]). The presence of many of these multi-PTMi spot containing proteins in critical cellular machineries (**Figure 4C**) and overall enrichment in heavily modified protein complexes suggests key roles for complex signal integration in human PTM signaling.

## Discussion

Large scale proteomic studies have provided high quality information of protein phosphorylation of functional cellular snapshots, different cell compartments, cell types or specific organs. Recently, the development of novel antibodies has enabled the mass spectrometry based, global investigation of lysine acetylation [20], [24] and ubiquitination [21]–[23] providing first views on thousands of these PTMs in human cell lines. However, these data have predominantly been analysed in isolation, even though PTM interplay is a crucial aspect for the function of key cellular players such as TP53, RNAPol II, EGFR or histones in the regulation of transcription and signaling. We postulated that ubiquitous PTMs such as pS/T, pY, Ac and Ub may be coordinated in cellular networks and addressed this through the generation of an integrated PTM map. More than 100,000 PTMs were mapped across ∼12,000 proteins and placed within a structured human protein interaction network, allowing these modifications to be put in context.

This initial map provided insight into two distinct mechanisms of PTM coordination across protein complexes; firstly, complexes as entireties accumulate modifications through preferential binding profiles suggesting coordination of single PTMs at the protein complex level (**Figure 1**). Through this protein complex targeting the cell can regulate specific molecular functions via a single trigger utilising one type of modification as a driver to mediate a given cellular response. This is supported by annotation analyses of PTM enriched complexes which revealed distinct, canonical functions for each modification (**Figure 1**). Interestingly, complex subunits that are highly modified by one PTM and thus contribute substantially to the modification enrichment across the complexes are also preferentially modified by other types of PTMs (**Figure 2**). This PTM coordination pattern was found prevalent across hundreds of protein complexes and yet had remained uncharacterized due to data paucity predicating systematic dataset integration. This mode of modification integration suggests a functional role in protein regulation, as it would allow multiple distinct regulatory systems to impact upon already highly targeted protein complexes.

Similar to our findings, it has been previously reported that protein complexes selectively accumulate acetylation modifications through preferential interaction profiles [24], yet here we show these complexes are also heavily modified by pS/T signaling. Exemplarily, Choudhary et al. highlight the NuRD chromatin remodeling complex as a novel target for high levels of lysine acetylation, while we find several distinct NuRD complexes that additionally contain >100 pS/T residues (**Dataset S2**). This suggests that while acetylation will almost certainly impact on molecular function, multiple inputs are likely required for stringent control of NuRD dependent transcriptional events. The prevalence of multiple PTM signals across all identified complexes would support the notion that greater biological insight can be gained through dataset integration, e.g. through prioritisation of highly, multiply modified components in further functional analyses.

A second layer of coordination was found through the preferential accumulation of proteins within these complexes that contain PTMi spots, integrating both single and multiple PTMs in short defined sequence spaces. We provide evidence to support the hypothesis that PTMi spots, i.e. dense clusters of modifications in defined amino acid sequence stretches, represent functional entities. We contrast PTMi spots to classically defined protein domains and suggest equal importance in normal cellular functioning (**Figure 3**).

Two recent reports into global PTM analysis used distinct measures to identify putatively functional PTMs. Beltrao et al. identified and characterized individual PTM “hot spots” present within certain protein domain families [27]. The approach presented here provides a conceptually different and thus complementary dataset focusing on clusters of PTMs located outside of protein domains that provide an alternate source of PTM regulation in the cell. Minguez et al. used co-evolution of reported PTM sites to infer a functional network of multiple PTMs, providing evidence that PTM signal integration, or “cross-talk”, is more wide spread than previously anticipated [49]. In addition, analyses presented in these reports support the hypothesis that PTMi spots represent functional entities. Beltrao et al. showed that phosphosites are more likely to be conserved, and therefore functionally relevant, when in proximity to modified lysines [27], while the fraction of co-evolving PTM sites identified by Minguez et al. is higher in PTMi spots (74%) than present across the entire PTM dataset (68%, dataset comparison details in Materials and Methods).

The presence of multiple PTM signals in short sequence stretches could provide exquisite sensitivity in PTM signaling, for example through multivalent interactions with other cellular components [47]. However simple combinatorial enumeration of all PTMi spot “states” makes clear that neither so many biochemically distinct species can simultaneously exist in a cell [50] nor that these states could be interpreted distinctively. Many of the PTMi spot states will be present in low fractional occupancies [35], [50] or be mutually exclusive. PTM systems have been proposed to require three tiers consisting of readers, writers and erasers for full functionality of a specific modification [51]. For any of these three components to function they must first recognise and bind the target protein sequence. The addition or removal of one or multiple modifications could directly affect the ability of other system components to bind to, and therefore impact upon, a given protein (or vice versa), therefore limiting the combinatorial output and allowing the coordination of multiple modifications in short sequence spaces.

The strengths and weaknesses of this analysis are inherent in the data collation. This integrated modification map significantly advances our understanding of PTM signaling, with the modifications collated here likely representing the more prevalent PTMs as predicted by the number of components encoded in the genome to regulate them. However, not only are these datasets likely incomplete, over 400 modifications impact on protein function within the cell [1], each with distinct temporal and spatial constraints. Furthermore, modifications such as protein methylation are thought to be widespread yet lack the proper tools for systematic investigation [52]. Therefore this map will increase in complexity as more systematic datasets arise for both the PTMs analysed here in distinct cellular context and for novel modifications. For example, POLR2A's C-terminal tail is annotated as modified by phosphorylation in this analysis, however it has recently been shown that methylation of R1810 interplays with proximal phosphorylation sites to allow correct functioning within the cell [13]. Methylation also interplays with phosphorylation signaling at the cell surface, negatively modulating EGFR signaling and influencing tumor model progression *in vivo* [15]. Furthermore, even less well characterized modifications such as O-Linked β-N-acetylglucosamine have also been shown to play a role in PTM interplay with phosphorylation histone H3 [53]. Therefore as these datasets become more comprehensive and functional protein complexes become more comprehensively defined, we predict our ability to identify sites of PTM interplay and cellular processes affected will increase.

Here we have shown two distinct mechanisms of signal integration exist within human protein complexes; firstly multiple signals are integrated on individual subunits of highly modified PTM complexes, predominantly in low density or spatially separated regions. However, these complexes are enriched for PTMi spots with the capacity to integrate multiple PTMs in short defined sequence spaces. PTMi spots likely represent functionally important regions within proteins on a proteome wide level. These emergent features of the PTM landscape within interaction datasets highlight the requisite for dataset integration in future HTP proteomic studies to gain a better understanding of the context of observed cellular signaling states.

## Materials and Methods

### Dataset collation and characterization

#### Post translational modifications

Data for each PTM was obtained from PhosphositePlus [54] and integrated with publically available datasets to obtain a non-redundant list of 13 amino acid sequences (13mers). The central amino acid is annotated as modified in each 13mer and only modified tyrosine, lysine, serine or threonine residues were taken forward to the final analysis. Each 13mer was standardised in annotation type to an individual RefSeq protein sequence. Identical proteins from distinct Entrez Gene IDs were collapsed onto a single identifier (For example SMN1 & 2 and also the calmodulins, **Dataset S1**). In the case of histone proteins where multiple very similar protein products are annotated across many RefSeqs and gene IDs, the most highly annotated protein in each class was taken forward for further analysis as representative of the protein sub-family. The most highly modified RefSeq annotated sequence for each gene ID was then utilised for further analysis giving a final dataset of 100,391 uniquely mapped 13mers annotated across 12,127 unique proteins (**Dataset S1**).

In analogy to protein-protein interaction data each PTM dataset showed an approximate scale free distribution highlighting the presence of modification hubs (**Figure S16A**). With the exception of acetylation, the frequency of modification generally increased with increased protein size (**Figure S16B–E**). Acetylated proteins showed no difference between distinct bins of protein size suggestive of highly stringent modification control. Approximately 59% of proteins are modified by more than one PTM (**Figure S17A**), with multiply modified proteins showing only a small tendency for increased protein length (**Figure S17B**). Protein length is not however directly predictive of either high or multiple PTM modification as equally sized proteins can be poorly modified for one modification but highly modified for another, while smaller proteins can be modified by multiple PTMs (For examples see **Figure S17C**).

#### Protein domain annotation

Domain annotation was downloaded from HPRD in tab delimited format (version 072010) with only the annotation taken forward for the specific RefSeq identifier utilised in the PTM analysis.

#### Amino acid structural disorder prediction

Each RefSeq protein sequence annotated for post translational modification was analysed using the Iupred disorder prediction software [55], at 0.5 as a cut-off to binarise each amino acid into ordered or disordered.

#### Cosmic mutation dataset

The data was obtained from the Wellcome Trust Sanger Institute Cancer Genome Project web site, http://www.sanger.ac.uk/genetics/CGP, v58_150312. Mutations were filtered for only protein coding or frame-shift mutations that would likely affect functional protein domains. These mutations were then mapped to the annotated RefSeq protein sequences with only mutated amino acids annotated in the same position as on the protein sequence taken forward for further analysis giving a high confidence dataset of 182,581 mutations.

#### Tumor suppressor/Oncogene annotation

The 1258 genes annotated as tumor suppressor/oncogene were obtained from Memorial Sloan-Kettering Cancer Center CancerGenes dataset [56]. The 1078 present in the PTM dataset are annotated in Dataset S1.

#### Protein complexes

Complexes were downloaded from ConsensusPathDB [33], containing both unique and family expanded complexes mapped to entrez gene IDs. Family expanded complexes are multiple similar complexes that each contain one of several homologous proteins that could not be differentiated through experimental identification. This data was then filtered to obtain only complexes that contained ≥3 unique subunits, with family expanded complexes collapsed onto one representative complex, taking forward the complex most highly annotated for PTMs. The final data included 4462 unique protein complexes. Complex subunit size distribution is similar to the total proteome showing a long tailed distribution with fewer very large proteins highlighting that while complex datasets currently contain more smaller proteins than represented in the whole proteome, the proteins in these complexes cover a large proportion of the range of protein sizes encoded in the human genome (**Figure S18A**). Protein frequency in the dataset shows a similar distribution to binary interaction networks, with most proteins present very few times and a minority showing a high frequency in the dataset (**Figure S18B**). Similar to protein length, while protein frequency in the dataset is not directly predictive of total modifications there is a slight positive correlation (**Figure S18C**).

### Data analysis

#### Protein complex analysis

Random protein complex dataset generation was untaken via PTM annotation permutation performed at the protein level, keeping all modifications across an identifier linked when shuffled. Annotation permutation was performed within 16 individual bins of approximately 200–350 identifiers, each bin contained proteins within the same size and complex dataset frequency quartiles to account for slight correlations observed in the dataset characterisation. 100 annotation randomised datasets were generated before collating all data together and ascertaining the data distribution for the total and median levels of the randomized complexes for each modification (heatmaps underlying plot in **Figures 1A–D**).

Gene Ontology functional analysis was undertaken using ConsensusPathPD over-representation analysis tool using gene ontology level 4 categories for both biological processes and molecular functions (P-Value cut-off <0.05). Only identifiers present in complexes that were enriched for the designated PTMs alone were taken forward to the GO analysis.

#### PTM enrichment score calculation

To observe the origin of the PTM enrichment in protein complexes a Z-Score was used as an approximation of signal enrichment for each dataset. The proteins in each group of complexes was first sub-divided into groups dependent on their number of distinct modifications (1_PTM, 2_PTMs, 3_PTMs or 4_PTMs), and the total signal for each modification calculated, giving 16 values for each enriched complex group. The same number of complexes as present in each group was then randomly sampled 1000 times from the whole protein complex dataset and the total signal for each modification was obtained. The randomly sampled data predominantly followed approximate normal distributions (**Figures S5, S6, S7, S8, S9**), therefore to obtain a value for signal enrichment we calculated the standard Z-score for each of the 16 values obtained for each group of complexes.

#### Single protein 20 amino acid (AA) window scanning analysis

20AA scanning across a protein sequence was undertaken in two different modes: Firstly, to obtain local maxima of PTM density, 20AA windows were calculated with 10AA overlay to be able to ascertain the maximum PTM density over any given sequence space. Local, non-overlapping PTM peaks were then calculated, taking the AA window position of the local peak as the first position the PTM maximum is calculated, discarding all other data points.

Secondly, to compare PTM density with other annotations across the whole protein length continuous, non-overlapping 20AA windows were utilised. High PTM density is classified as a density of >0.3, medium PTM density as >0.05 and ≤0.3, and low as 0.05. For domain annotation 1–10AA overlap of a 20AA window with a protein domain was classified as a small overlap, 11–19AA a large overlap and 20AA as internal to a protein domain. For disordered amino acid prediction, the ratio of disordered residues in a 20AA window (x) was binned as follows; 0.5>x>0 representing low disorder, 1>x≥0.5 representing medium levels of disorder and 1 representing high levels of disorder. For cosmic mutations, the number of mutations annotated in a 20AA window was calculated prior to binarising as described in the main text.

Random PTM permutation analysis was performed on individual protein sequences keeping the type, frequency and density of modifications across the protein sequence constant, only shuffling the specific PTM annotation at each residue. This represents the most stringent randomisation analysis to determine if distinct modifications are in proximity solely through accumulation of PTMs in defined sequence spaces.

#### Short linear motif (SLIM) analysis

An enrichment analysis of short linear motifs in the PTMi spots was undertaken using the SLIMfinder software [41] with default settings.

#### Comparison with the PTMcode dataset

From the PTMcode dataset [49] we mapped 69,049 lysine acetylation, tyrosine or serine/threonine phosphorylation and lysine ubiquitination PTMs to 9,749 unique RefSeqs protein identifiers using mappings from the STRING database resource [57]. Of these modifications, 46,359 (∼67.1%) were annotated as co-evolving with at least one other PTM. The proportion of co-evolving PTMs that are also annotated in our dataset is roughly equivalent; from our dataset of 100,391 PTMs: 56,772 of these modifications were accurately mapped in the Minguez dataset, 38,970 (68.6%) of which are annotated as co-evolving. Minguez et al. predicted functional associations between PTMs based on a co-evolution measure. In support of the hypothesis that the clusters of PTMs highlighted here represent short sequence space to integrated multiple functional PTM signals, a higher percentage of PTMs are annotated as co-evolving (74%) in the Minguez dataset (2602 of the 3515 PTMs annotated here are mapped in the Minguez dataset, of which 1929 (74%) are annotated as co-evolving).

## Supporting Information

Dataset S1 **Collated post translation modification dataset.**
(XLSX)

Dataset S2 **Annotated protein complex dataset.**
(XLSX)

Dataset S3 **Dataset of local PTM density across individual proteins.**
(RAR)

Dataset S4 **Annotated PTMi spots.**
(XLSX)

Figure S1 **Protein complex data distribution for each PTM.** 2D density distribution for total and median modification of each complex for each PTM. For each plot blue to black regions represents increasing number of complexes in the dataset with the 500 most outlying protein complexes plotted as individual data points. Data points may be overlaid preventing visualisation of each complex values. 2D density distribution plot for (A) acetylation, (B) tyrosine phosphorylation, (C) ubiquitination and (D) serine/Threonine phosphorylation. Both x- and y-axis for (D) have been truncated for ease of visualisation excluding extreme outliers.
(PDF)

Figure S2 **Characterisation of PTM enriched complex size.** The two outlying groups of complexes were characterized for both total protein length and number of unique subunits present; (A) acetylation, (B) tyrosine phosphorylation, (C) ubiquitination and (D) serine/threonine phosphorylation. Cut-offs were utilised to generally characterize both sets of subgroups and are indicated in the top corner of each box plot.
(PDF)

Figure S3 **Schematic representation of network randomisation workflow.** Each unique protein is first binned based upon its frequency in the protein complex dataset. The proteins in each bin are then further divided into 4 sub-bins based on the protein size. Finally the annotations for the ∼200–350 proteins in each of these 16 separate bins are randomized.
(PDF)

Figure S4 **Large scale figure for complex-subgroup functional enrichment.** As for figure 1F but with GO term labels on the Y-axis. GO analysis highlighting coordinated differential molecular function control by each group of highly modified complexes. Ac & Ub represents the 57 complexes that are enriched for both these PTMs, ≥3PTMs represents the 39 complexes enriched in at least 3 PTMs.
(PDF)

Figure S5 **Signal enrichment determination for acetylation enriched complexes.** (Addendum to Main Figure 2C). The total signal obtained for each of 1000 random datasets displayed as histograms. For each random dataset the number of complexes randomly sampled was equal to the number of complexes present in each sub group in the real dataset. The value in the real dataset is indicated with an arrow.
(PDF)

Figure S6 **Signal enrichment determination for phospho-tyrosine enriched complexes.** (Addendum to Main Figure 2C). The total signal obtained for each of 1000 random datasets displayed as histograms. For each random dataset the number of complexes randomly sampled was equal to the number of complexes present in each sub group in the real dataset. The value in the real dataset is indicated with an arrow.
(PDF)

Figure S7 **Signal enrichment determination for ubiquitination enriched complexes.** (Addendum to Main Figure 2C). The total signal obtained for each of 1000 random datasets displayed as histograms. For each random dataset the number of complexes randomly sampled was equal to the number of complexes present in each sub group in the real dataset. The value in the real dataset is indicated with an arrow.
(PDF)

Figure S8 **Signal enrichment determination for phospho-serine or phospho-threonine enriched complexes.** (Addendum to Main Figure 2C). The total signal obtained for each of 1000 random datasets displayed as histograms. For each random dataset the number of complexes randomly sampled was equal to the number of complexes present in each sub group in the real dataset. The value in the real dataset is indicated with an arrow.
(PDF)

Figure S9 **Signal enrichment determination for complexes enriched in 3 or more PTMs.** (Addendum to Main Figure 2C). The total signal obtained for each of 1000 random datasets displayed as histograms. For each random dataset the number of complexes randomly sampled was equal to the number of complexes present in each sub group in the real dataset. The value in the real dataset is indicated with an arrow.
(PDF)

Figure S10 **Modifications in 20AA windows.** (A) The number of non-overlapping PTM density peaks (identified with 20AA windows) associated with each combination of modifications present across the proteins in the enriched complexes. (B) The percentage of signal for each modification in high density PTM windows in the enriched complexes in comparison to all windows over the complex dataset. (C) The distribution of distinct PTM density local peaks across proteins within the PTM enriched complex data.
(PDF)

Figure S11 **PTM window mutational analysis.** (A) Frequency of 20AA windows across a protein sequence that are frequently mutated in cancerous cells, based on their protein domain annotation (coloured bars). The frequency of 20AA windows outside of protein domain annotations that are frequently mutated in cancerous cells, based on their PTM density (Grey bars). In contrast to Main Figure 3F, here a cut-off of >2 mutations/20 AA window was utilised to binarise with respect to cancer association. (B) Mutated 20AA window analysis in A however using a cut-off of >1 mutation/20 AA window and restricting the dataset to only complex components annotated as oncogenes or tumour suppressor genes [56].
(PDF)

Figure S12 **Distribution of PTMi spots across the protein complexes.** Bar-chart showing the percentage of complexes with PTMi spots across either the entire complex dataset or the PTM enriched complexes.
(PDF)

Figure S13 **PTMi spots identification and characterization across the modified proteome.** Same analysis as presented in Main Figure 3 but across the entire PTM dataset. (A) 2D density plot of local STYK density windows plotted against local PTM density with a histogram of number of local peaks. The 500 most outlying data points are plotted as points on the density plot, however with substantial overlay preventing visualisation. (B) 2D density plot of the number of 20 AA windows containing more than 1 PTM. (C) The number of high density windows that contain multiple PTMs compared to 100 random annotation permutation simulations. (D) Overlap analysis between individual PTM 20AA windows and annotated protein domains. Int: 20AA window internal to a protein domain, Lg: Large overlap with a proteins domain (>10AAs), Sm: Small overlap with a protein domain, Ext: 20AA window external to an annotated protein domain. (E) Overlap analysis between individual PTM 20AA windows and predicted protein disorder. High: Every amino acid is predicted to be disordered, Med: 11–19 AAs in a window are predicted to be disordered, Low: 1–10 AAs in a window are predicted to be disordered. (F) Frequency of 20AA windows across a protein sequence that are mutated in cancerous cells, sorted based on their protein domain annotation (coloured bars). The frequency of 20AA windows outside of annotated protein domains that are mutated in cancerous cells, sorted based on their PTM density (Grey bars). (G) Mutated 20AA widows analysis as in F however restricting the dataset to genes annotated as oncogenes or tumour suppressor genes.
(PDF)

Figure S14 **Proteome wide STYK ratio, disorder content and PTM density analysis.** (A) Upper panel: Bar graph; Total number of 20AA windows spread across all proteins above a local STYK density of 0.3. Red line; Total number of medium or high disordered 20AA windows at each STYK density. Lower panel: Percentage of total 20AA windows in each disorder bin at a given STYK ratio. (B) PTM density distribution of the modified windows at a given STYK density. (C) Upper panel: Bar graph; Total number of 20AA windows with a PTM density of >0.3 at each STYK density of 0.3. Lower panel: Percentage of high PTM density 20AA windows in each disorder bin at a given STYK ratio.
(PDF)

Figure S15 **PY-NLS sequences present in PTMi spots.** Top: Amino acid sequence of the canonical PY-NLS present in HNRNPA1. The tripartite NLS motif is highlighted with transparent boxes over the protein sequence. pS residues annotated in this analysis and known to reduce karyopherinβ affinity highlighted. Bottom: Four PY-NLS sequences present in PTMi spots in this analysis. Modified amino acids and the tripartite motifs surrounding the NLS sequence highlighted.
(PDF)

Figure S16 **Individual PTM dataset analysis.** (A) Log:Log Distribution of each individual PTM data. Box plots of protein modifications binned by protein size for (B) acetylation, (C) tyrosine phosphorylation, (D) ubiquitination, (E) serine/threonine phosphorylation and (F) total PTMs. Y-axes are truncated to exclude outliers for ease of visualisation. Size ranges are approximately equal bins representing small (<297AAs), med-small (298–494AAs), med-large (494–757AAs) and large proteins (>757AAs).
(PDF)

Figure S17 **Basic overlap PTM dataset analysis.** (A) Overlap analysis of proteins shown to be modified by each individual PTM. (B) Box plots of total protein length binned by number of distinct PTM modifications, y axis has been truncated to 10,000 AAs for ease of visualisation. (C) Three tables providing examples of group of proteins that are equal in size together with the number of distinct modifications as examples for specific PTM states.
(PDF)

Figure S18 **Complex subunit dataset analysis.** (A) Histogram of protein length present in the protein complex dataset (black bars) compared to all PTM proteins (white bars). (B) Histogram of subunit frequency in the protein complex dataset. (C) Box plot of total PTM modification binned by frequency in the protein complex dataset, Y axis has been truncated to 100 modifications for ease of visualisation.
(PDF)
