# Supplementary material for: Dual Coordination of Post Translational Modifications in Human Protein Networks
Source: PLoS Comput Biol. 2013 Mar 7;9(3):e1002933. doi: 10.1371/journal.pcbi.1002933 (PMC3591266; doi:10.1371/journal.pcbi.1002933)
Supplement: Figure S1 — Protein complex data distribution for each PTM. 2D density distribution for total and median modification of each complex for each PTM. For each plot blue to black regions represents increasing number of complexes in the dataset with the 500 most outlying protein complexes plotted as individual data points. Data points may be overlaid preventing visualisation of each complex values. 2D density distribution plot for (A) acetylation, (B) tyrosine phosphorylation, (C) ubiquitination and (D) serine/Threonine phosphorylation. Both x- and y-axis for (D) have been truncated for ease of visualisation excluding extreme outliers. (PDF) [file pcbi.1002933.s005.pdf]

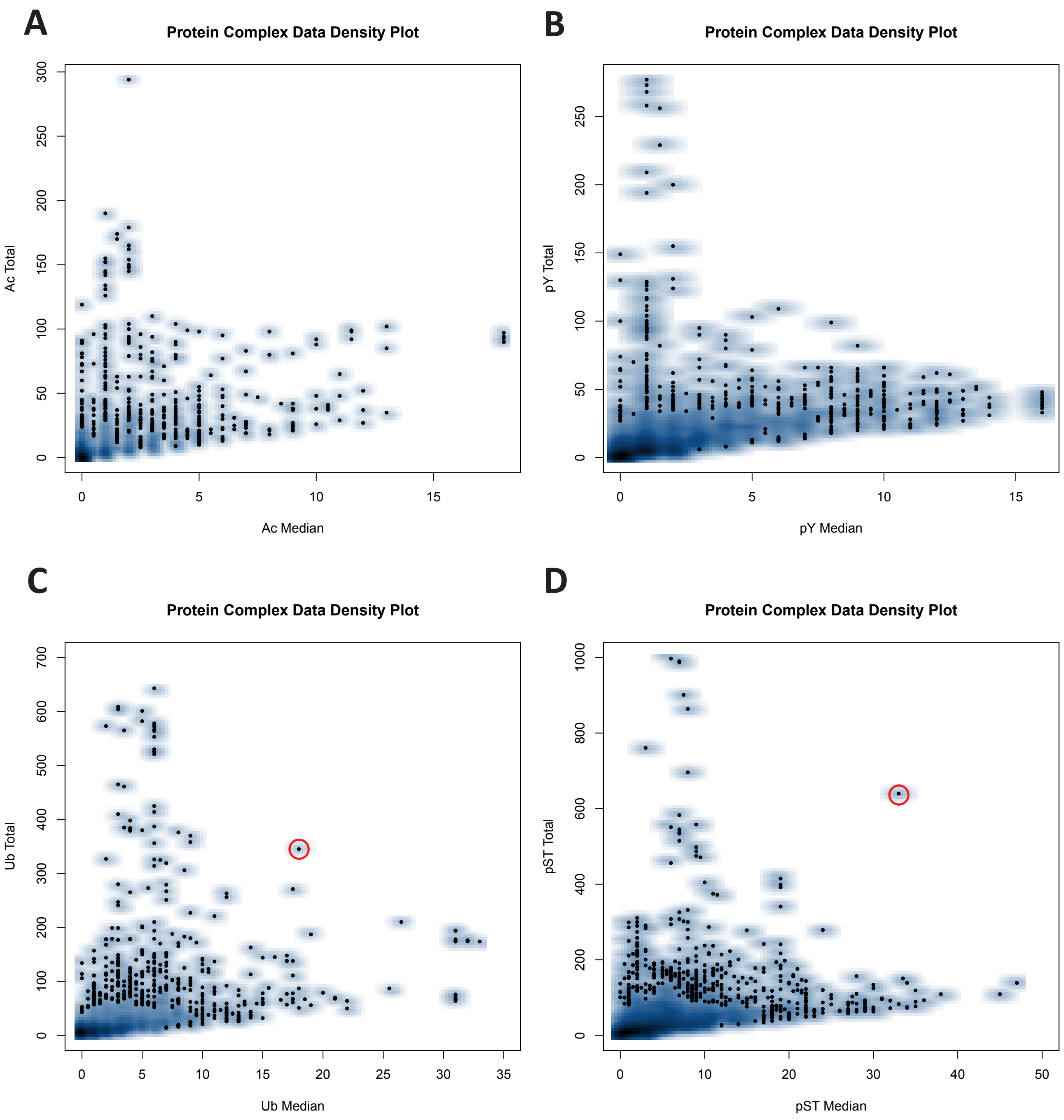
A
Protein Complex Data Density Plot
Ac Total
Ac Median
B
Protein Complex Data Density Plot
pY Total
pY Median
C
Protein Complex Data Density Plot
Ub Total
Ub Median
D
Protein Complex Data Density Plot
pST Total
pST Median
