# Supplementary figures and images for: Dual Coordination of Post Translational Modifications in Human Protein Networks
Source: PLoS Comput Biol. 2013 Mar 7;9(3):e1002933. doi: 10.1371/journal.pcbi.1002933 (PMC3591266; doi:10.1371/journal.pcbi.1002933)

**A**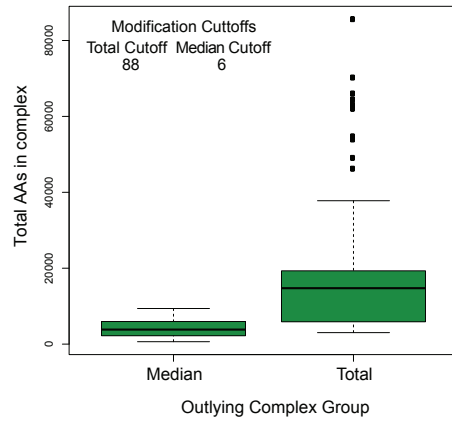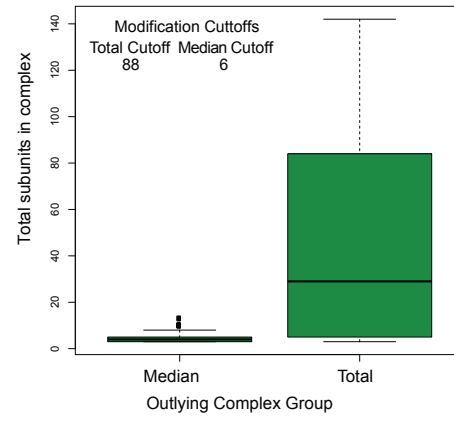**B**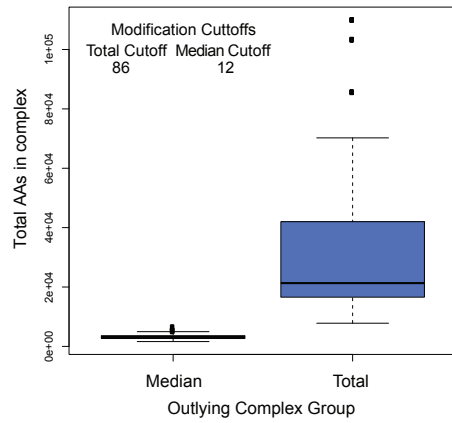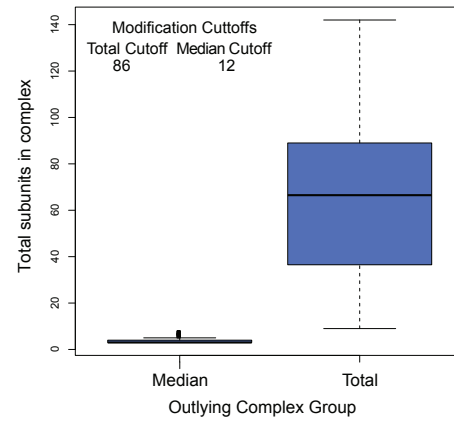**C**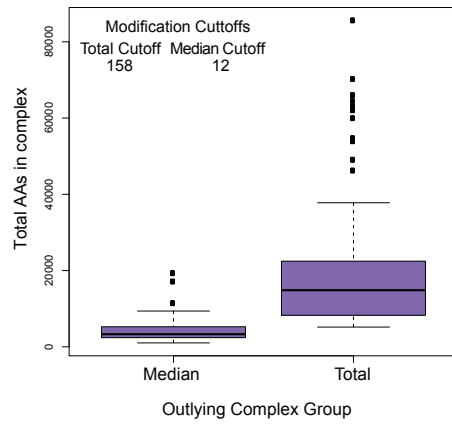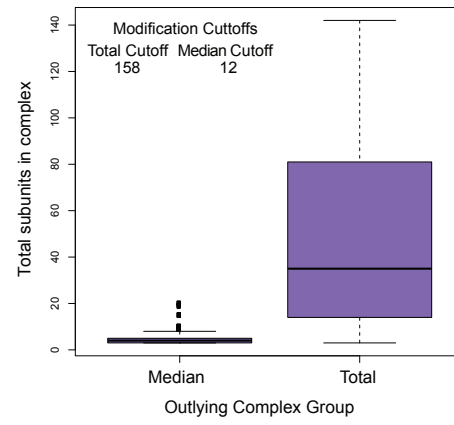**D**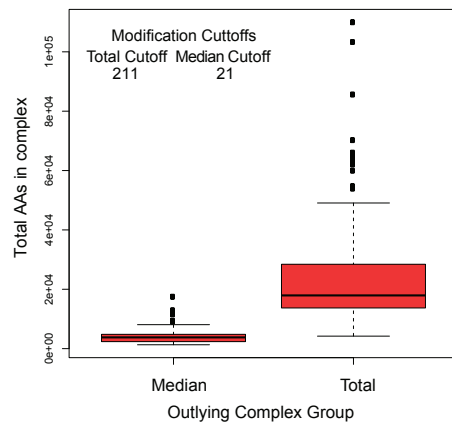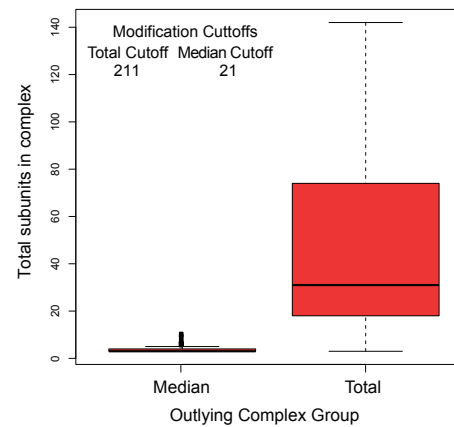

Supplement: Figure S2 — Characterisation of PTM enriched complex size. The two outlying groups of complexes were characterized for both total protein length and number of unique subunits present; (A) acetylation, (B) tyrosine phosphorylation, (C) ubiquitination and (D) serine/threonine phosphorylation. Cut-offs were utilised to generally characterize both sets of subgroups and are indicated in the top corner of each box plot. (PDF) [file pcbi.1002933.s006.pdf]

**4462 Protein Complexes:  
4143 unique proteins**

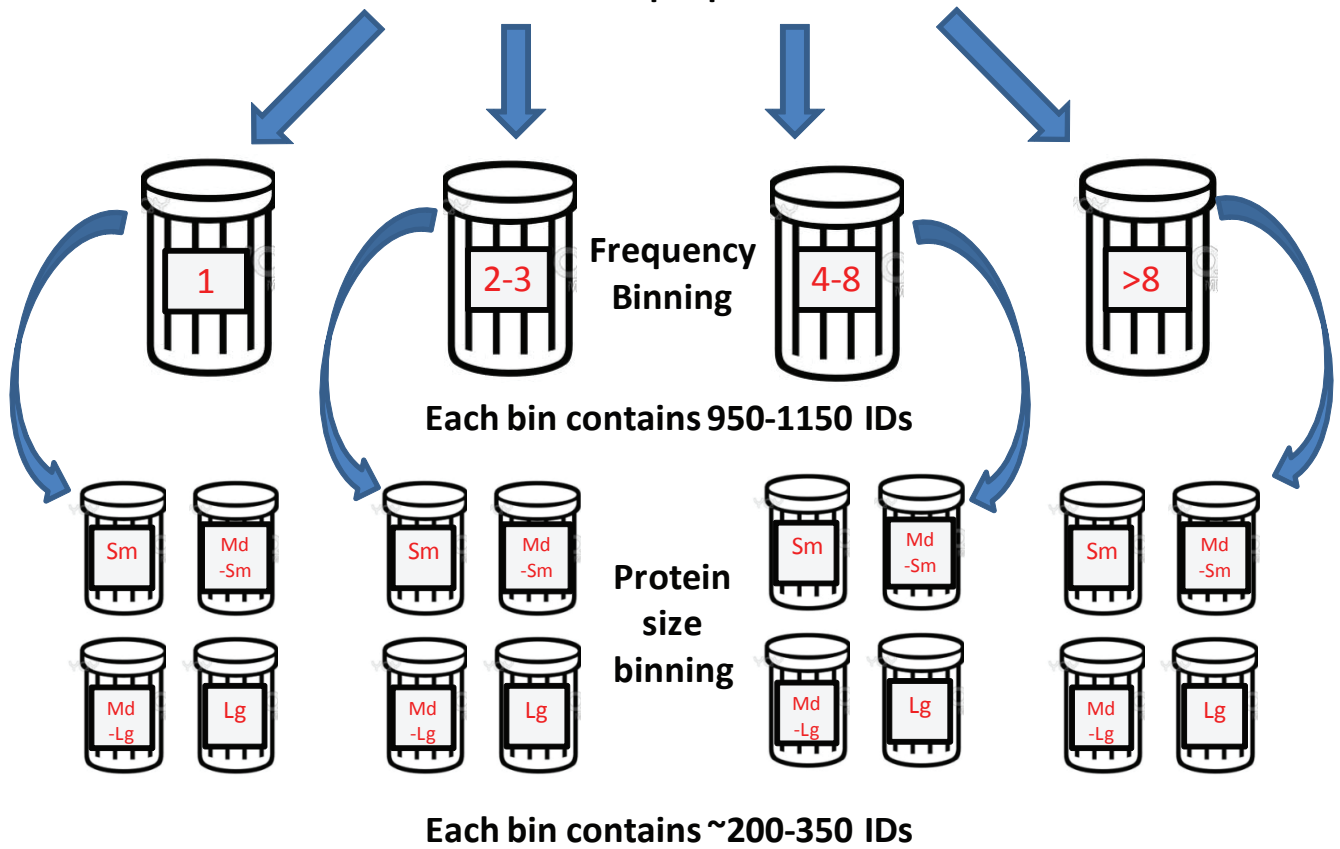

Supplement: Figure S3 — Schematic representation of network randomisation workflow. Each unique protein is first binned based upon its frequency in the protein complex dataset. The proteins in each bin are then further divided into 4 sub-bins based on the protein size. Finally the annotations for the ∼200–350 proteins in each of these 16 separate bins are randomized. (PDF) [file pcbi.1002933.s007.pdf]

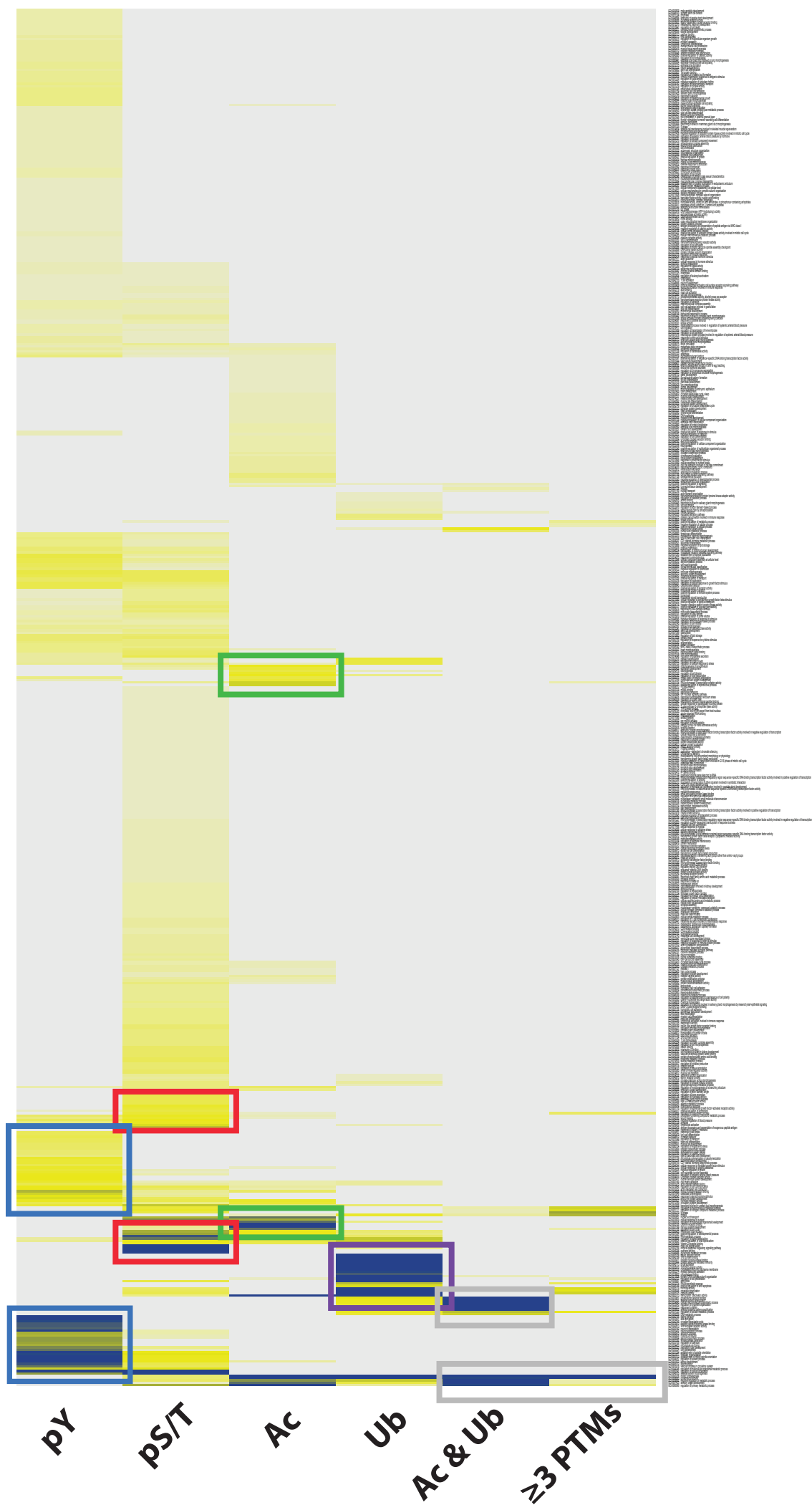

Supplement: Figure S4 — Large scale figure for complex-subgroup functional enrichment. As for figure 1F but with GO term labels on the Y-axis. GO analysis highlighting coordinated differential molecular function control by each group of highly modified complexes. Ac & Ub represents the 57 complexes that are enriched for both these PTMs, ≥3PTMs represents the 39 complexes enriched in at least 3 PTMs. (PDF) [file pcbi.1002933.s008.pdf]

Ac Selected Complexes

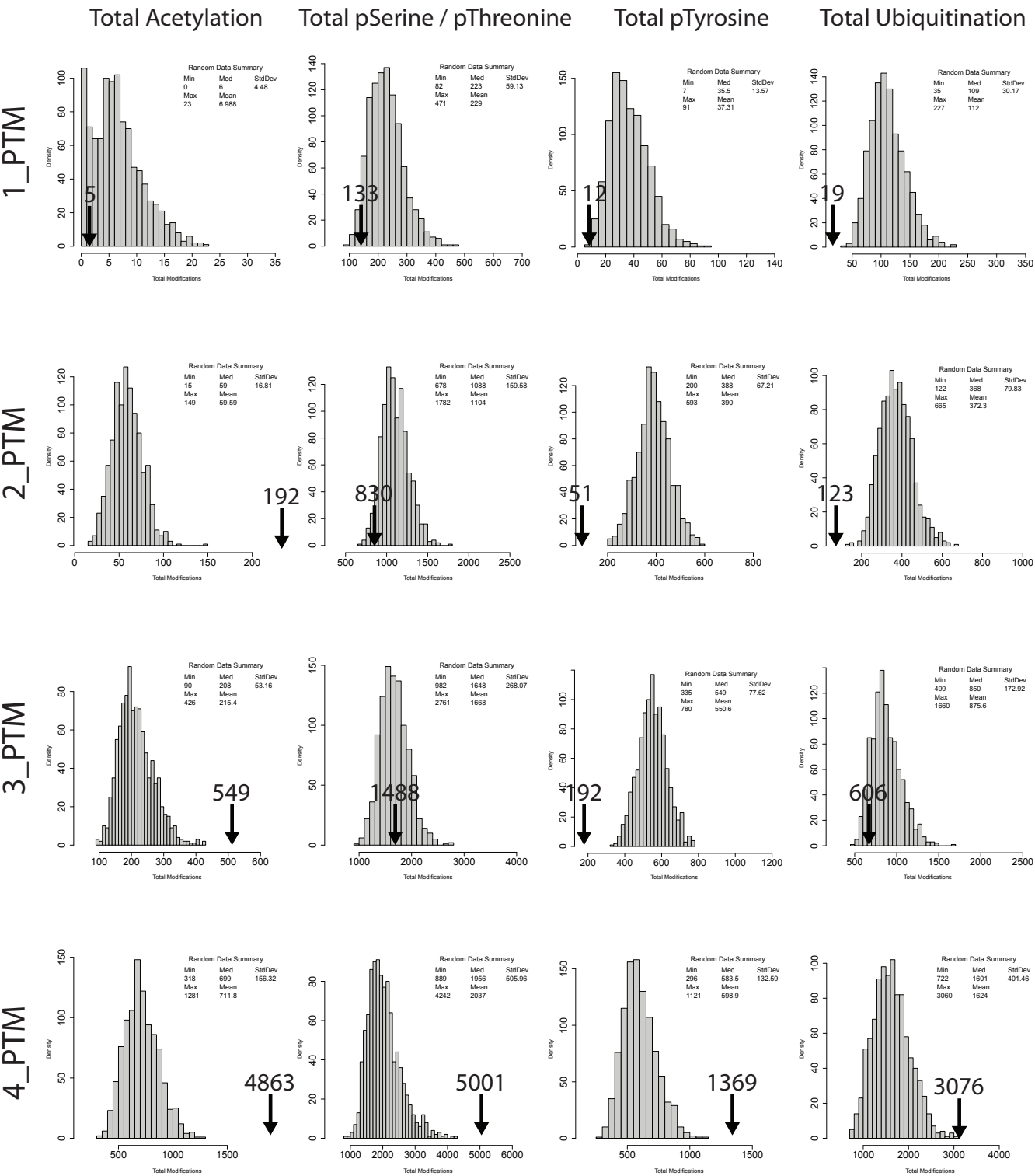

Supplement: Figure S5 — Signal enrichment determination for acetylation enriched complexes. (Addendum to Main Figure 2C). The total signal obtained for each of 1000 random datasets displayed as histograms. For each random dataset the number of complexes randomly sampled was equal to the number of complexes present in each sub group in the real dataset. The value in the real dataset is indicated with an arrow. (PDF) [file pcbi.1002933.s009.pdf]

pY Selected Complexes

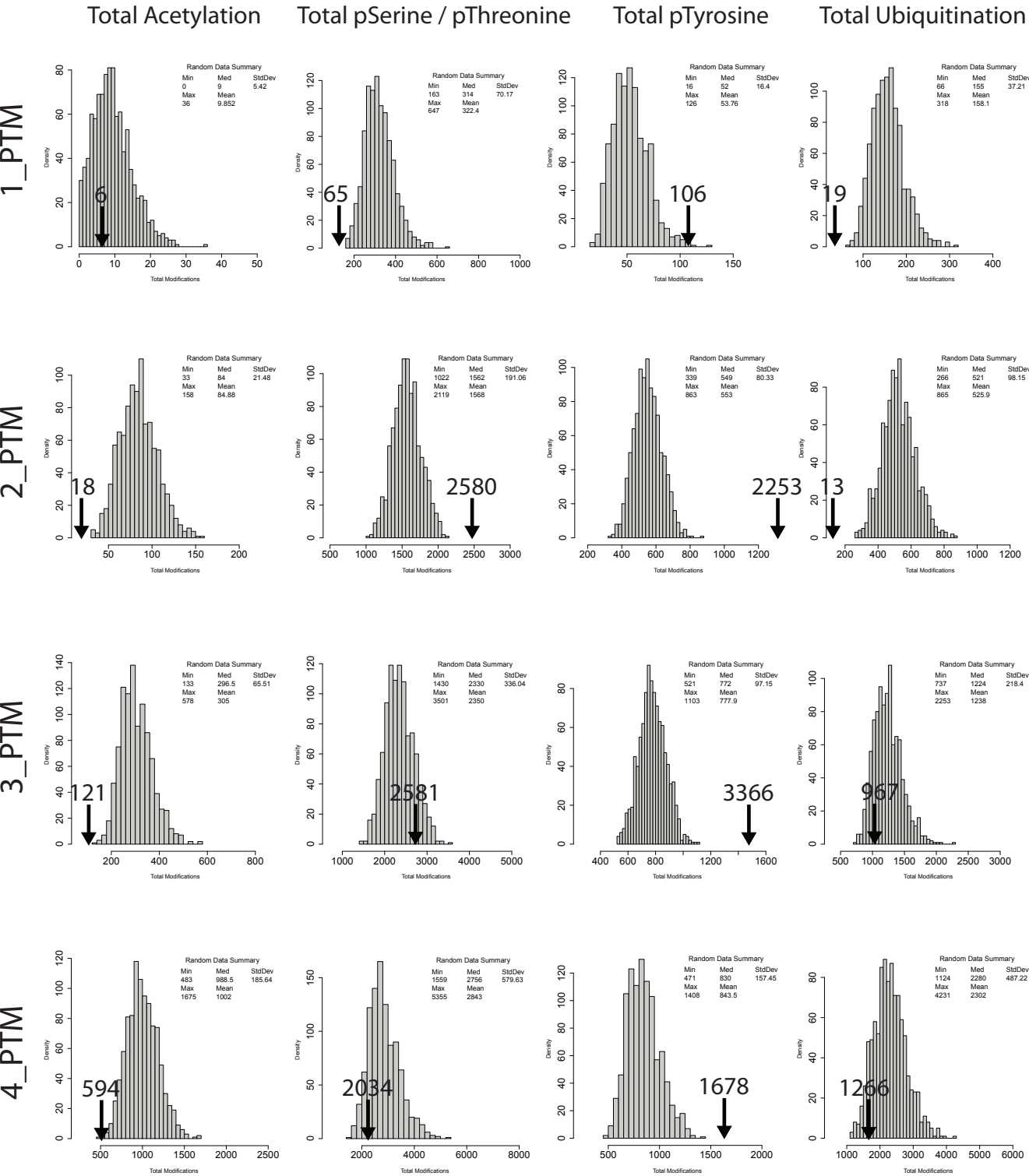

Supplement: Figure S6 — Signal enrichment determination for phospho-tyrosine enriched complexes. (Addendum to Main Figure 2C). The total signal obtained for each of 1000 random datasets displayed as histograms. For each random dataset the number of complexes randomly sampled was equal to the number of complexes present in each sub group in the real dataset. The value in the real dataset is indicated with an arrow. (PDF) [file pcbi.1002933.s010.pdf]

Ub Selected Complexes

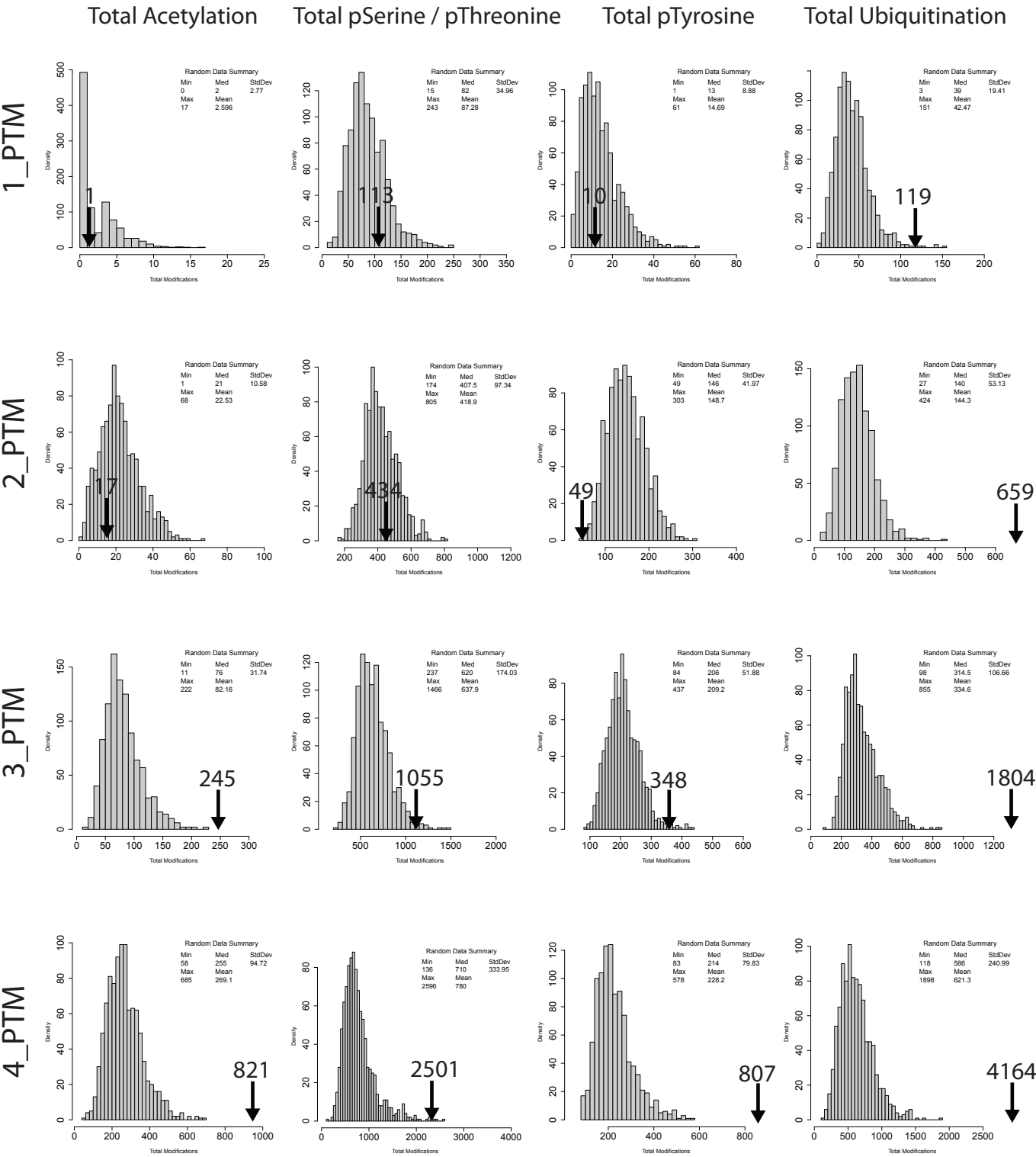

Supplement: Figure S7 — Signal enrichment determination for ubiquitination enriched complexes. (Addendum to Main Figure 2C). The total signal obtained for each of 1000 random datasets displayed as histograms. For each random dataset the number of complexes randomly sampled was equal to the number of complexes present in each sub group in the real dataset. The value in the real dataset is indicated with an arrow. (PDF) [file pcbi.1002933.s011.pdf]

pS / pT Selected Complexes

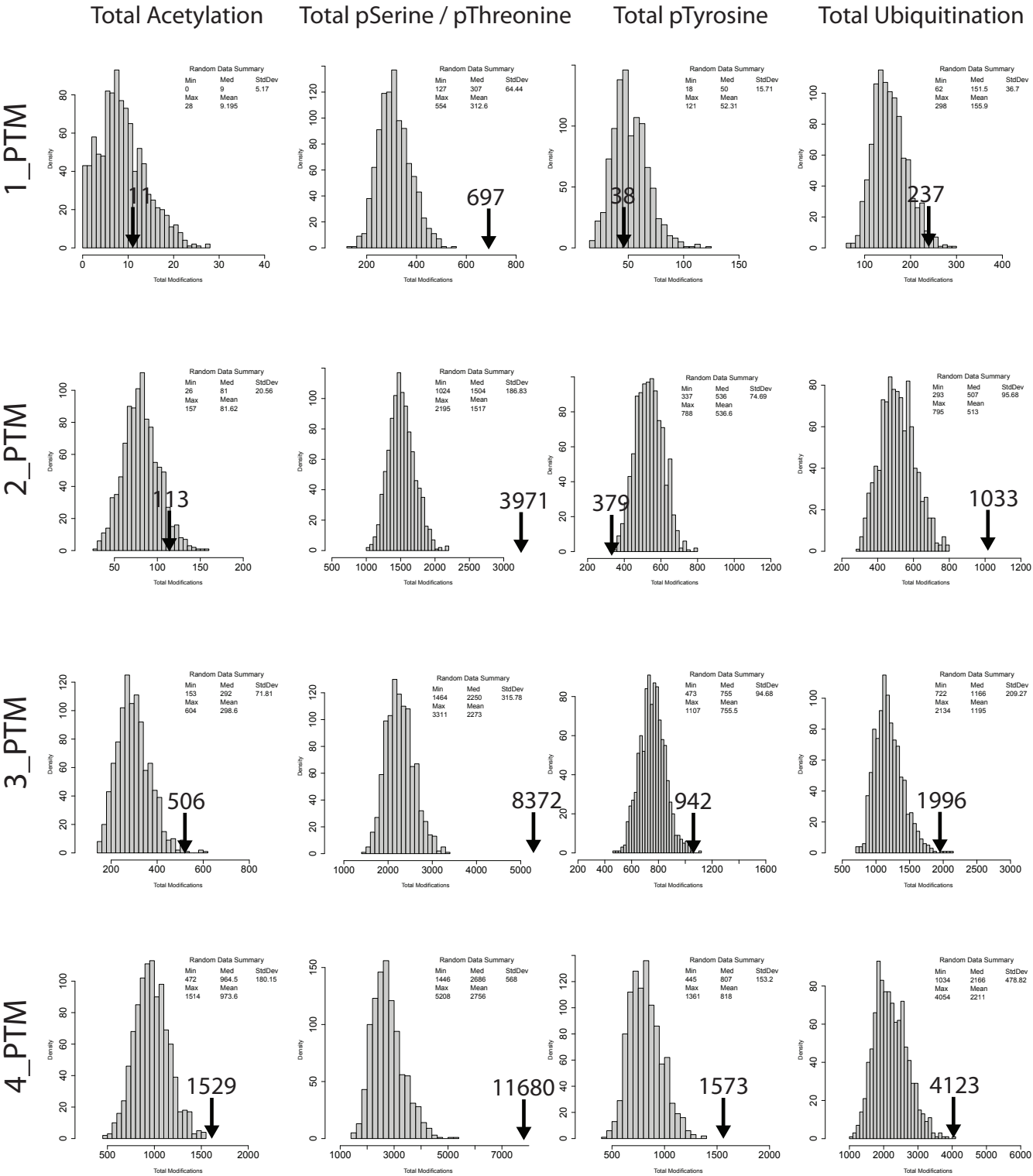

Supplement: Figure S8 — Signal enrichment determination for phospho-serine or phospho-threonine enriched complexes. (Addendum to Main Figure 2C). The total signal obtained for each of 1000 random datasets displayed as histograms. For each random dataset the number of complexes randomly sampled was equal to the number of complexes present in each sub group in the real dataset. The value in the real dataset is indicated with an arrow. (PDF) [file pcbi.1002933.s012.pdf]

### 3 or more PTMs Selected Complexes

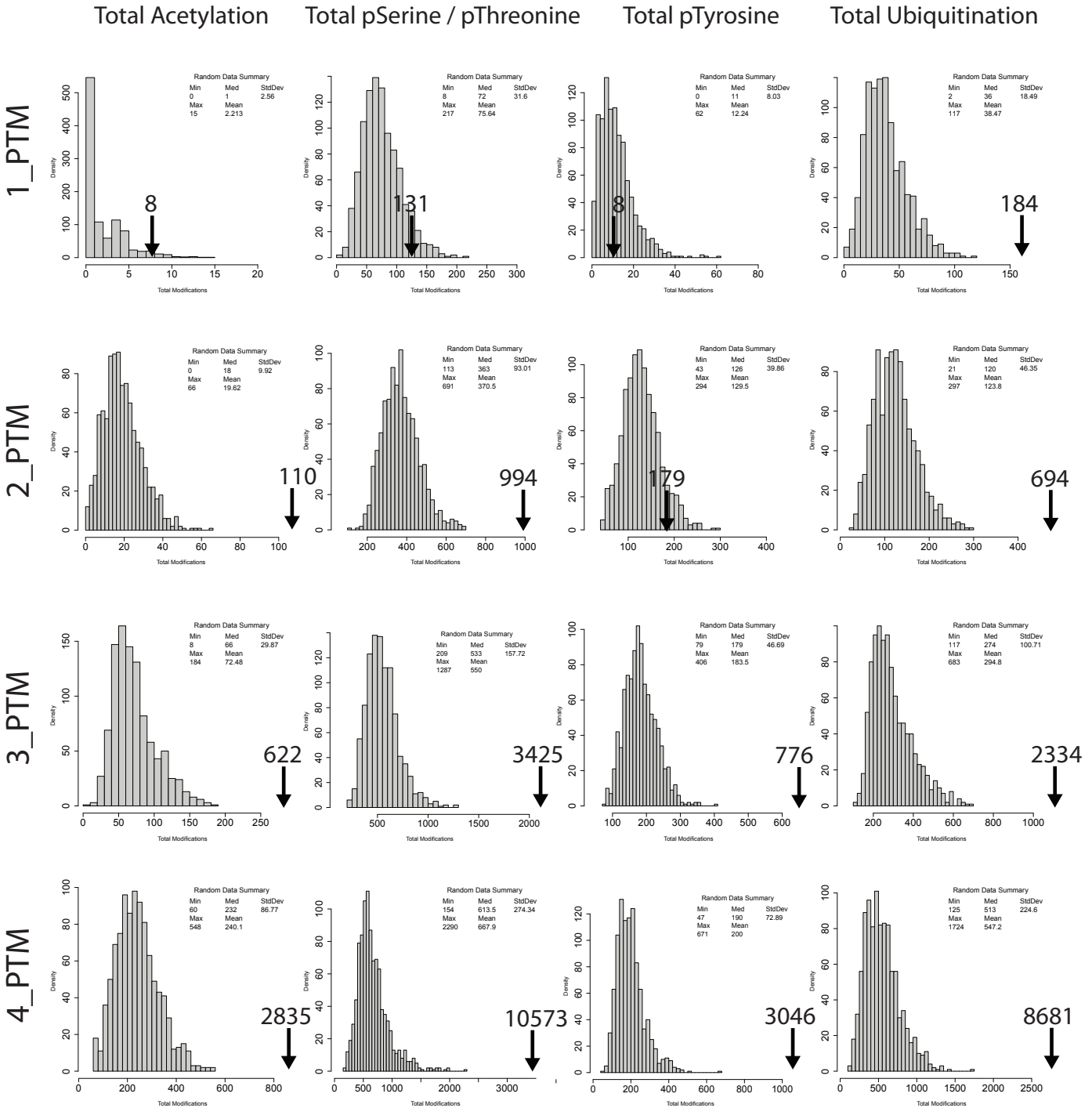

Supplement: Figure S9 — Signal enrichment determination for complexes enriched in 3 or more PTMs. (Addendum to Main Figure 2C). The total signal obtained for each of 1000 random datasets displayed as histograms. For each random dataset the number of complexes randomly sampled was equal to the number of complexes present in each sub group in the real dataset. The value in the real dataset is indicated with an arrow. (PDF) [file pcbi.1002933.s013.pdf]

**A**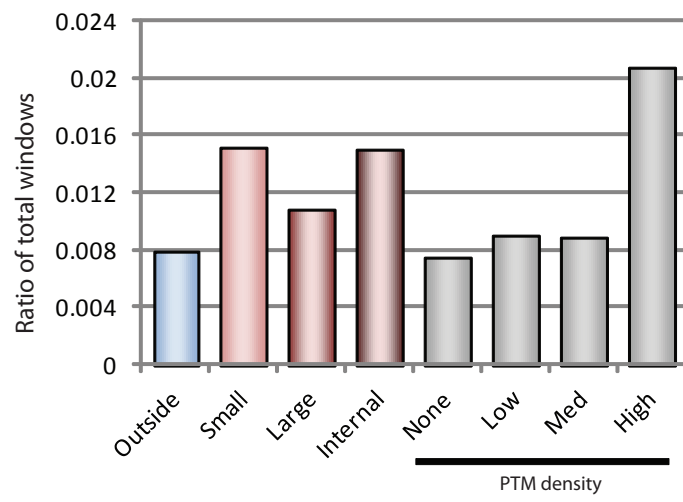**B**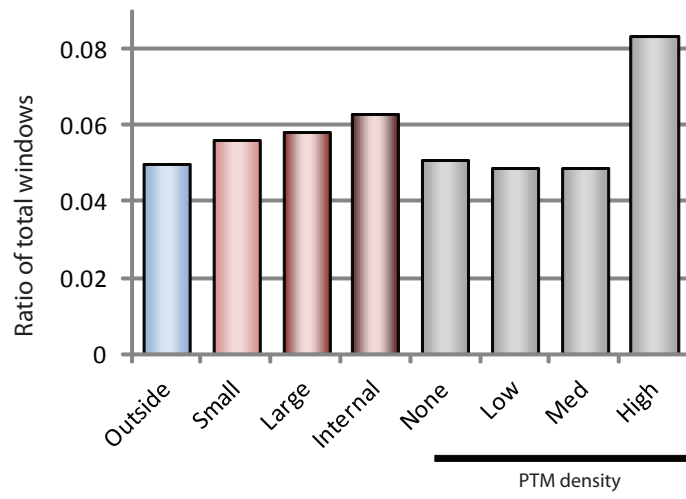

Supplement: Figure S11 — PTM window mutational analysis. (A) Frequency of 20AA windows across a protein sequence that are frequently mutated in cancerous cells, based on their protein domain annotation (coloured bars). The frequency of 20AA windows outside of protein domain annotations that are frequently mutated in cancerous cells, based on their PTM density (Grey bars). In contrast to Main Figure 3F, here a cut-off of >2 mutations/20 AA window was utilised to binarise with respect to cancer association. (B) Mutated 20AA window analysis in A however using a cut-off of >1 mutation/20 AA window and restricting the dataset to only complex components annotated as oncogenes or tumour suppressor genes [56]. (PDF) [file pcbi.1002933.s015.pdf]

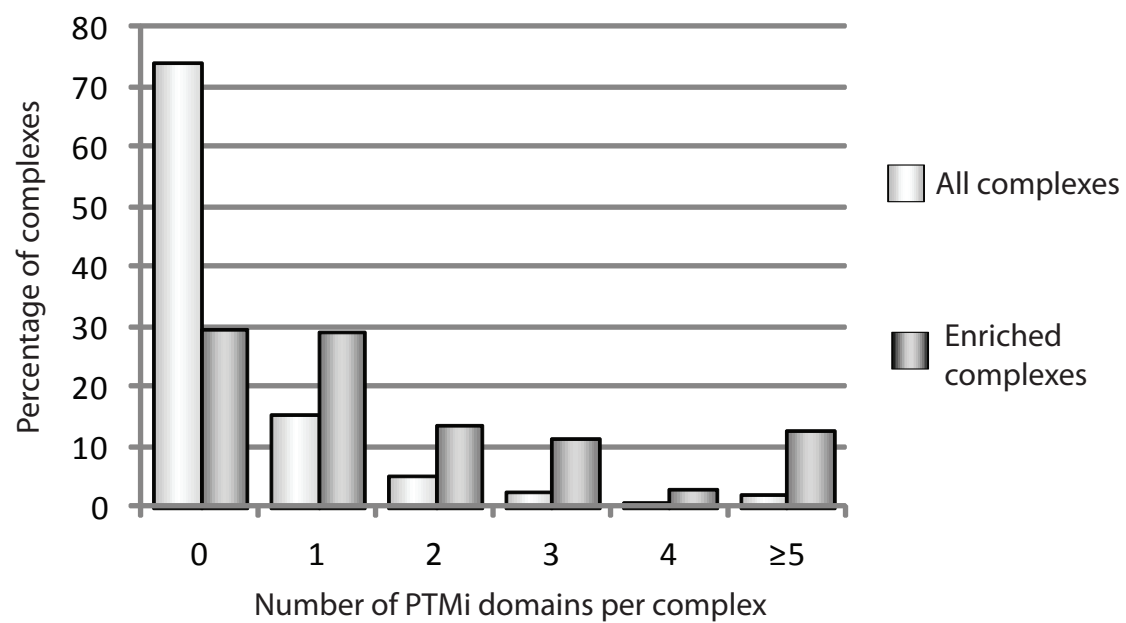

Supplement: Figure S12 — Distribution of PTMi spots across the protein complexes. Bar-chart showing the percentage of complexes with PTMi spots across either the entire complex dataset or the PTM enriched complexes. (PDF) [file pcbi.1002933.s016.pdf]

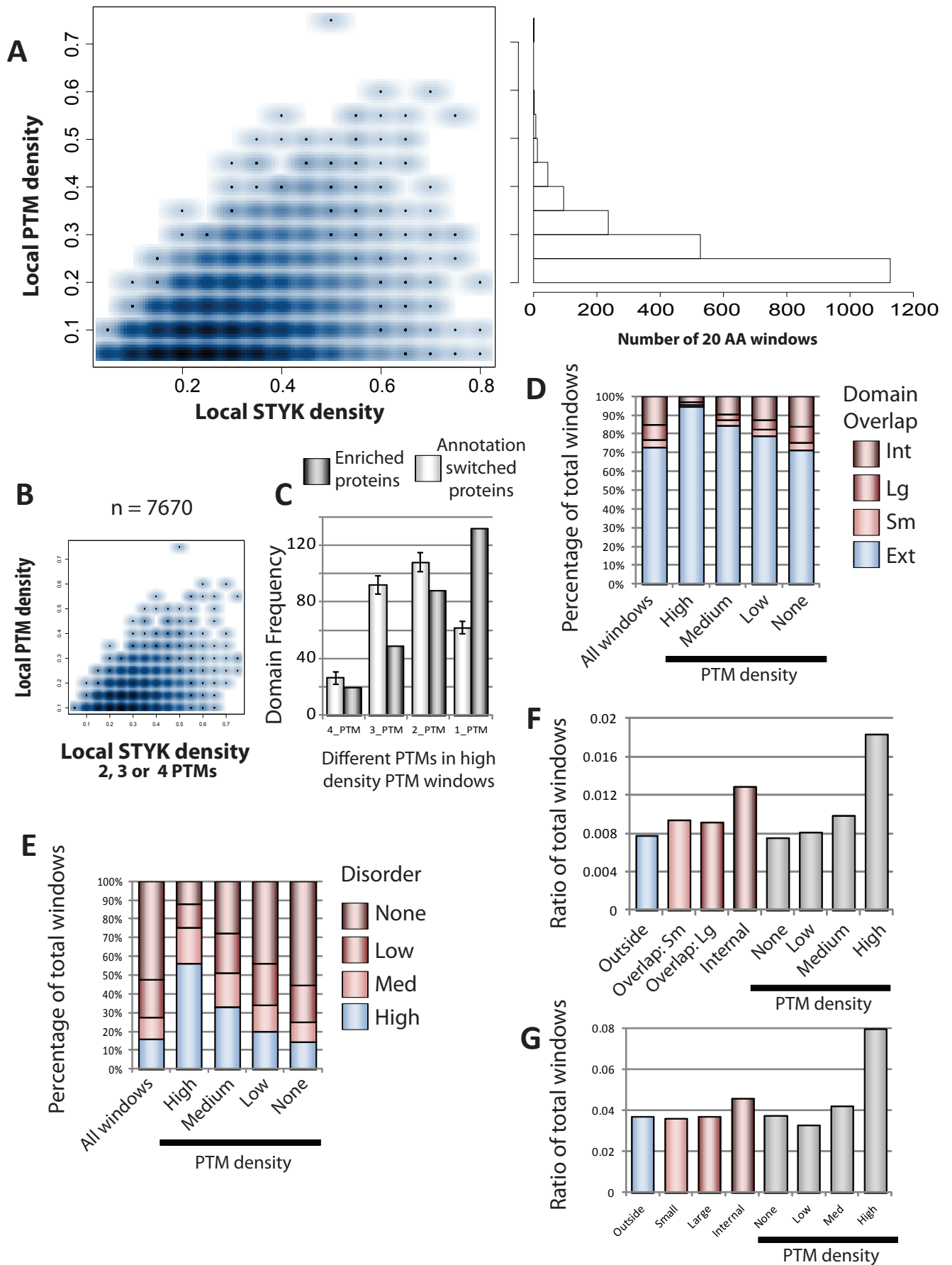

Supplement: Figure S13 — PTMi spots identification and characterization across the modified proteome. Same analysis as presented in Main Figure 3 but across the entire PTM dataset. (A) 2D density plot of local STYK density windows plotted against local PTM density with a histogram of number of local peaks. The 500 most outlying data points are plotted as points on the density plot, however with substantial overlay preventing visualisation. (B) 2D density plot of the number of 20 AA windows containing more than 1 PTM. (C) The number of high density windows that contain multiple PTMs compared to 100 random annotation permutation simulations. (D) Overlap analysis between individual PTM 20AA windows and annotated protein domains. Int: 20AA window internal to a protein domain, Lg: Large overlap with a proteins domain (>10AAs), Sm: Small overlap with a protein domain, Ext: 20AA window external to an annotated protein domain. (E) Overlap analysis between individual PTM 20AA windows and predicted protein disorder. High: Every amino acid is predicted to be disordered, Med: 11–19 AAs in a window are predicted to be disordered, Low: 1–10 AAs in a window are predicted to be disordered. (F) Frequency of 20AA windows across a protein sequence that are mutated in cancerous cells, sorted based on their protein domain annotation (coloured bars). The frequency of 20AA windows outside of annotated protein domains that are mutated in cancerous cells, sorted based on their PTM density (Grey bars). (G) Mutated 20AA widows analysis as in F however restricting the dataset to genes annotated as oncogenes or tumour suppressor genes. (PDF) [file pcbi.1002933.s017.pdf]

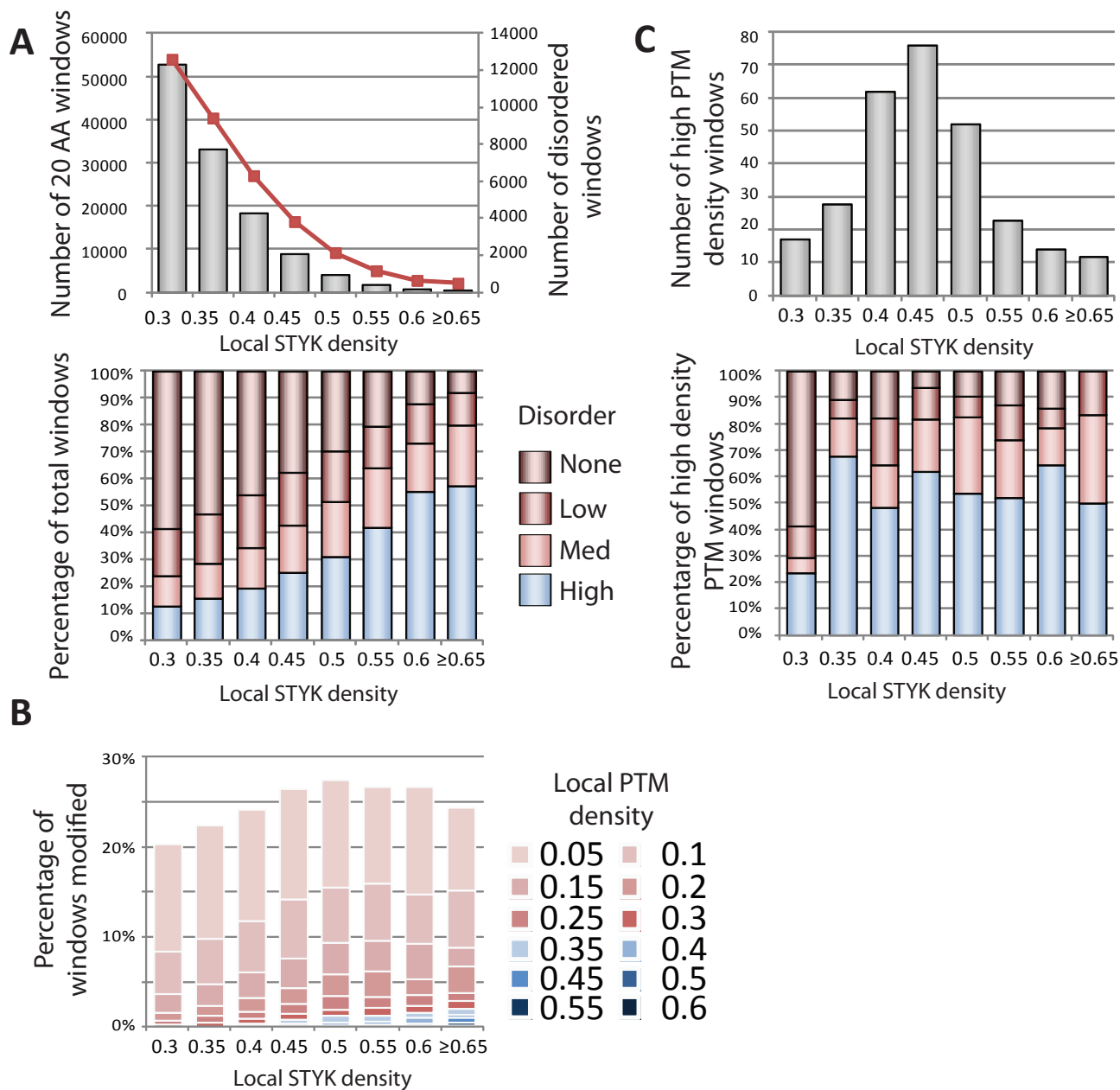

Supplement: Figure S14 — Proteome wide STYK ratio, disorder content and PTM density analysis. (A) Upper panel: Bar graph; Total number of 20AA windows spread across all proteins above a local STYK density of 0.3. Red line; Total number of medium or high disordered 20AA windows at each STYK density. Lower panel: Percentage of total 20AA windows in each disorder bin at a given STYK ratio. (B) PTM density distribution of the modified windows at a given STYK density. (C) Upper panel: Bar graph; Total number of 20AA windows with a PTM density of >0.3 at each STYK density of 0.3. Lower panel: Percentage of high PTM density 20AA windows in each disorder bin at a given STYK ratio. (PDF) [file pcbi.1002933.s018.pdf]

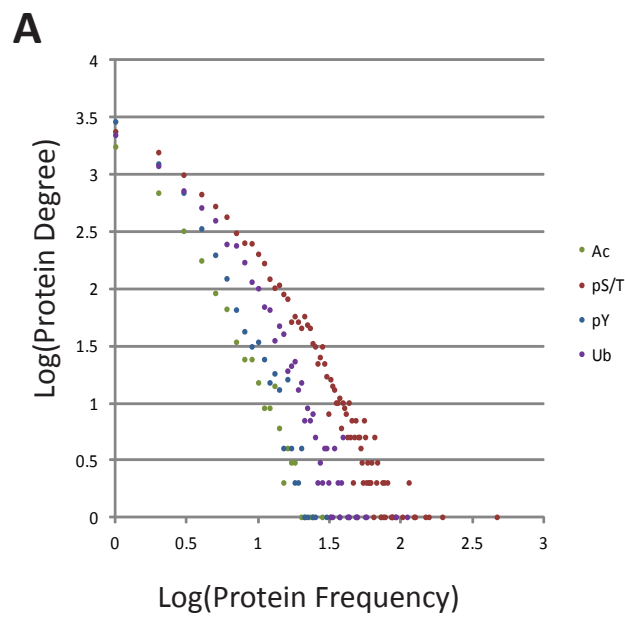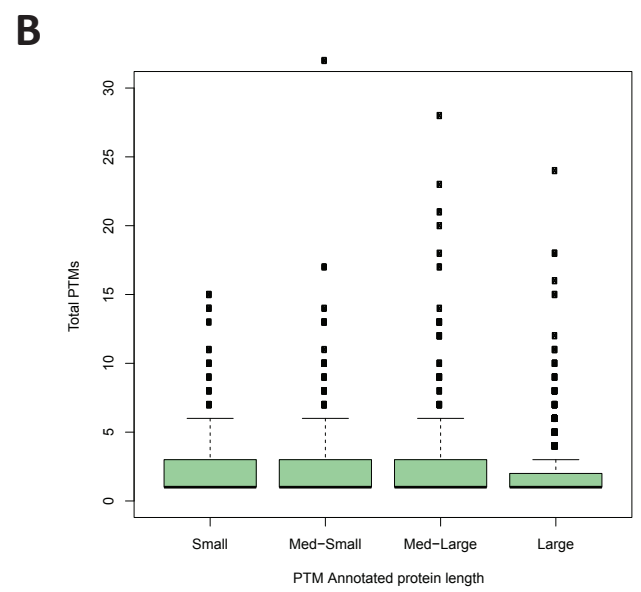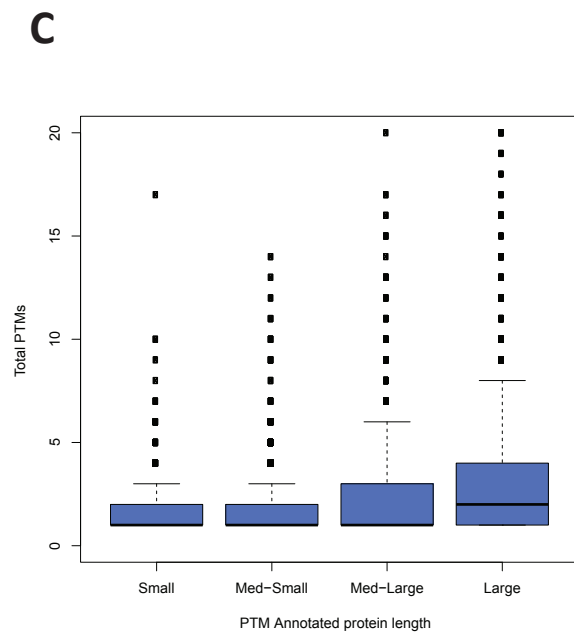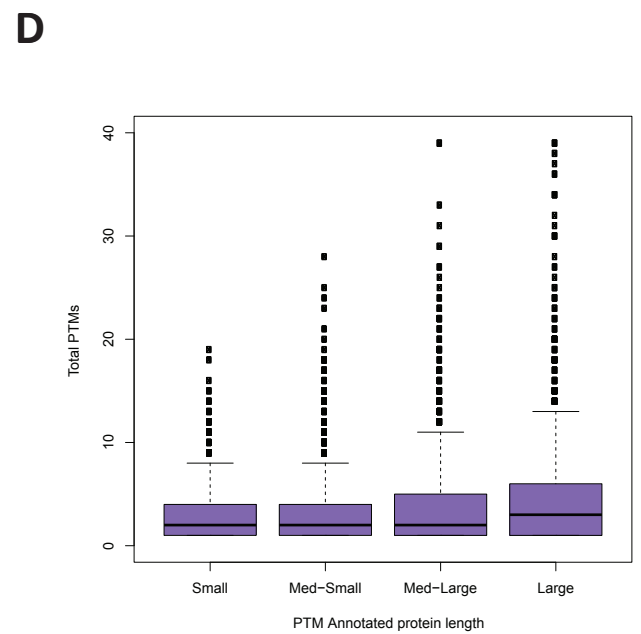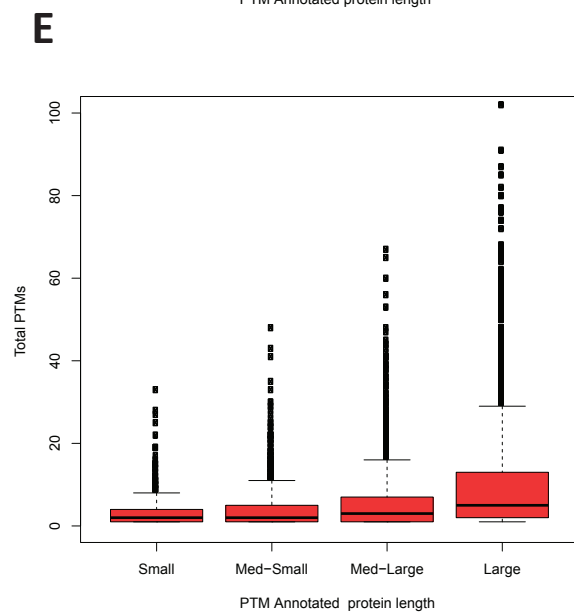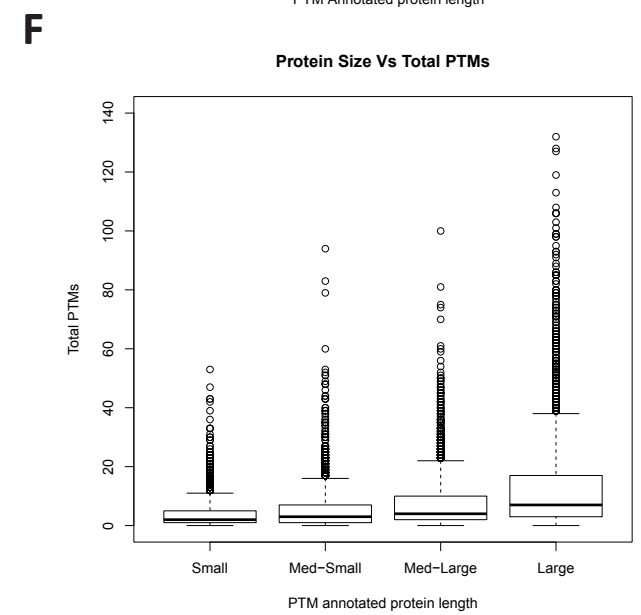

Supplement: Figure S16 — Individual PTM dataset analysis. (A) Log:Log Distribution of each individual PTM data. Box plots of protein modifications binned by protein size for (B) acetylation, (C) tyrosine phosphorylation, (D) ubiquitination, (E) serine/threonine phosphorylation and (F) total PTMs. Y-axes are truncated to exclude outliers for ease of visualisation. Size ranges are approximately equal bins representing small (<297AAs), med-small (298–494AAs), med-large (494–757AAs) and large proteins (>757AAs). (PDF) [file pcbi.1002933.s020.pdf]
