# Supplementary material for: Dual Coordination of Post Translational Modifications in Human Protein Networks
Source: PLoS Comput Biol. 2013 Mar 7;9(3):e1002933. doi: 10.1371/journal.pcbi.1002933 (PMC3591266; doi:10.1371/journal.pcbi.1002933)

A

	PTM Density		
	High	Med	Low
pS/T	87	2176	1813
pY	0	249	744
Ub	0	611	1731
Ac	2	102	351
pS/T & Ac	6	156	0
Ac & Ub	6	448	0
pS/T & Ub	13	621	0
pS/T & pY	39	624	0
pY & Ub	0	235	0
pY & Ac	0	46	0
pS/T & Ub & Ac	25	157	0
pS/T & pY & Ac	1	18	0
pS/T & pY & Ub	8	114	0
pY & Ub & Ac	2	100	0
pS/T & pY & Ac & Ub	18	58	0
**Multiple Modifications (%)**	**57.0**	**45.1**	**0.00**

B

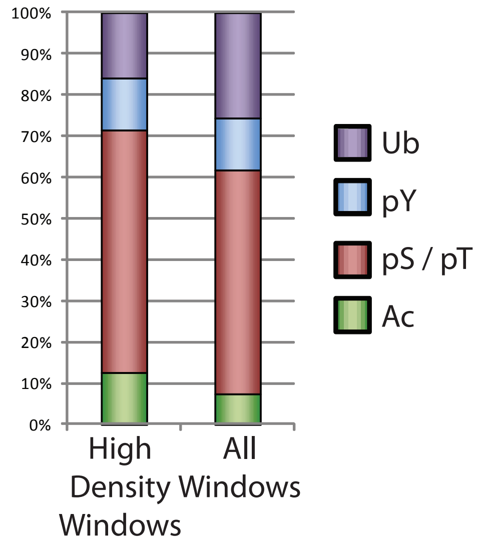

C

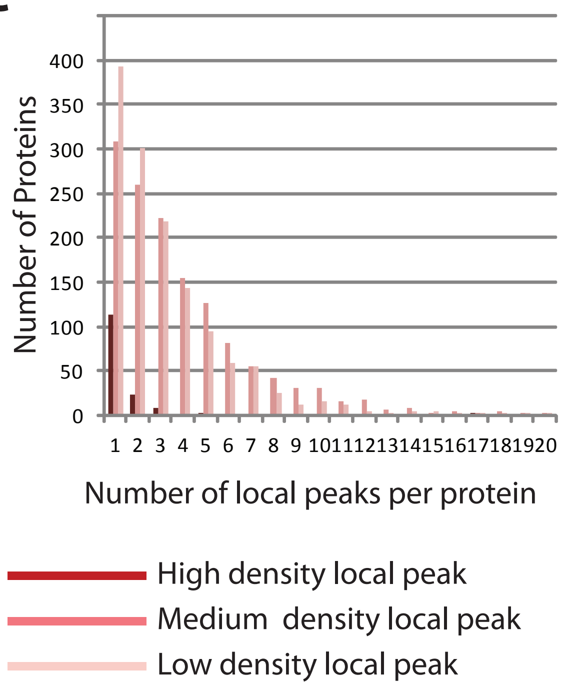

Supplement: Figure S10 — Modifications in 20AA windows. (A) The number of non-overlapping PTM density peaks (identified with 20AA windows) associated with each combination of modifications present across the proteins in the enriched complexes. (B) The percentage of signal for each modification in high density PTM windows in the enriched complexes in comparison to all windows over the complex dataset. (C) The distribution of distinct PTM density local peaks across proteins within the PTM enriched complex data. (PDF) [file pcbi.1002933.s014.pdf]
