# Supplementary material for: Dual Coordination of Post Translational Modifications in Human Protein Networks
Source: PLoS Comput Biol. 2013 Mar 7;9(3):e1002933. doi: 10.1371/journal.pcbi.1002933 (PMC3591266; doi:10.1371/journal.pcbi.1002933)
Supplement: Figure S15 — PY-NLS sequences present in PTMi spots. Top: Amino acid sequence of the canonical PY-NLS present in HNRNPA1. The tripartite NLS motif is highlighted with transparent boxes over the protein sequence. pS residues annotated in this analysis and known to reduce karyopherinβ affinity highlighted. Bottom: Four PY-NLS sequences present in PTMi spots in this analysis. Modified amino acids and the tripartite motifs surrounding the NLS sequence highlighted. (PDF) [file pcbi.1002933.s019.pdf]

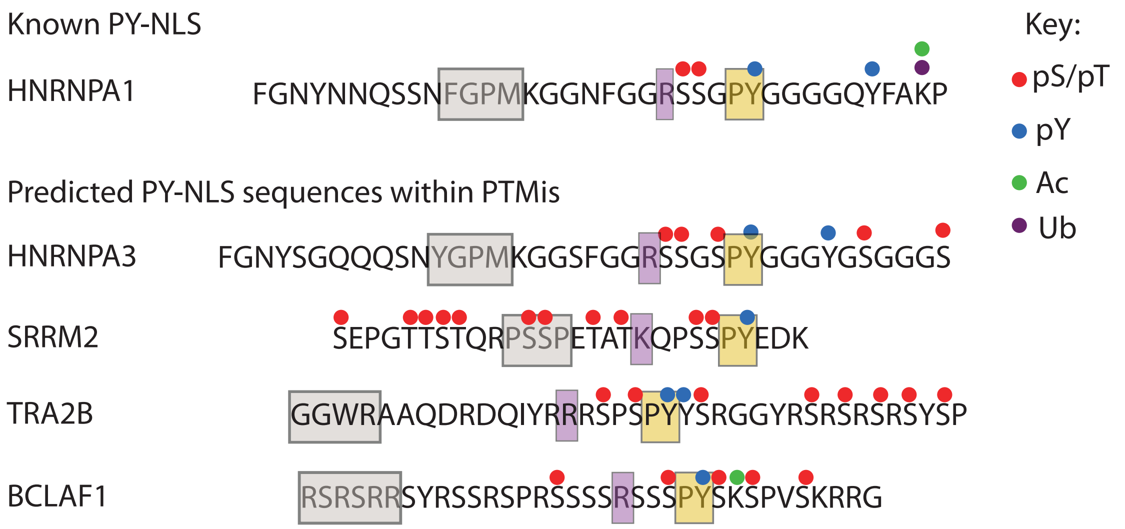
Known PY-NLS
HNRNPA1
FGNYNNQSSNFGPMKGGNFGGRSSGPYGGGGQYFAKP
Predicted PY-NLS sequences within PTMis
HNRNPA3
FGNYSGQQQSNYGPMKGGSFGGRSSGSPYGGGYGSGGGS
SRRM2
SEPGTTSTQRPSSPETATKQPSSPYEDK
TRA2B
GGWRAAQDRDQIYRRRSPSPYYSRGGYRSRSRSRSYSP
BCLAF1
RSRSRRSYRSSRSPRSSSSRSSSPYSKSPVSKRRG
Key:
pS/pT
pY
Ac
Ub
