# Supplementary material for: Dual Coordination of Post Translational Modifications in Human Protein Networks
Source: PLoS Comput Biol. 2013 Mar 7;9(3):e1002933. doi: 10.1371/journal.pcbi.1002933 (PMC3591266; doi:10.1371/journal.pcbi.1002933)
Supplement: Figure S17 — Basic overlap PTM dataset analysis. (A) Overlap analysis of proteins shown to be modified by each individual PTM. (B) Box plots of total protein length binned by number of distinct PTM modifications, y axis has been truncated to 10,000 AAs for ease of visualisation. (C) Three tables providing examples of group of proteins that are equal in size together with the number of distinct modifications as examples for specific PTM states. (PDF) [file pcbi.1002933.s021.pdf]

A

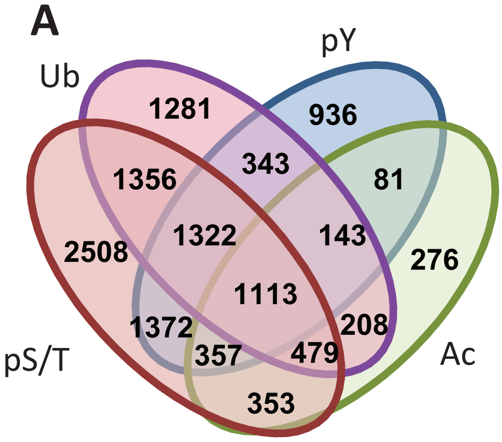

B

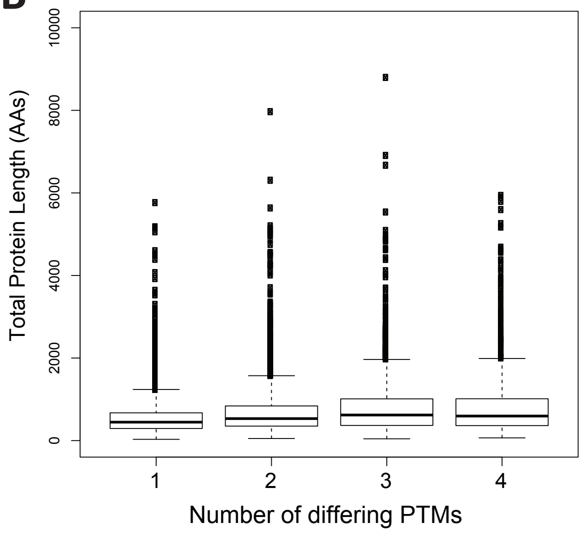

C

Large proteins with distinct modification number and patterns								
**Symbol**	**EGID**	**Acc**	**pS/T**	**pY**	**Ub**	**PTM_Total**	**Length**	**Diff_PTM_Freq**
HMCN1	83872	1	1	0	0	2	5635	2
MDN1	23195	1	13	6	43	63	5596	4
MLL2	8085	11	45	3	0	59	5537	3

Medium sized proteins with distinct modification number and patterns								
**Symbol**	**EGID**	**Acc**	**pS/T**	**pY**	**Ub**	**PTM_Total**	**Length**	**Diff_PTM_Freq**
DDR1	780	0	2	10	2	14	919	3
LIG1	3978	0	26	0	4	30	919	2
HMGCR	3156	0	1	0	19	20	888	2
ENPEP	2028	0	0	2	0	2	957	1

Small, multiply modified proteins								
**Symbol**	**EGID**	**Acc**	**pS/T**	**pY**	**Ub**	**PTM_Total**	**Length**	**Diff_PTM_Freq**
TMSB10	9168	4	4	0	4	12	44	3
RPS29	6235	1	1	1	2	5	67	4
HMGN2	3151	15	2	0	3	20	90	3
