# Supplementary material for: Dual Coordination of Post Translational Modifications in Human Protein Networks
Source: PLoS Comput Biol. 2013 Mar 7;9(3):e1002933. doi: 10.1371/journal.pcbi.1002933 (PMC3591266; doi:10.1371/journal.pcbi.1002933)
Supplement: Figure S18 — Complex subunit dataset analysis. (A) Histogram of protein length present in the protein complex dataset (black bars) compared to all PTM proteins (white bars). (B) Histogram of subunit frequency in the protein complex dataset. (C) Box plot of total PTM modification binned by frequency in the protein complex dataset, Y axis has been truncated to 100 modifications for ease of visualisation. (PDF) [file pcbi.1002933.s022.pdf]

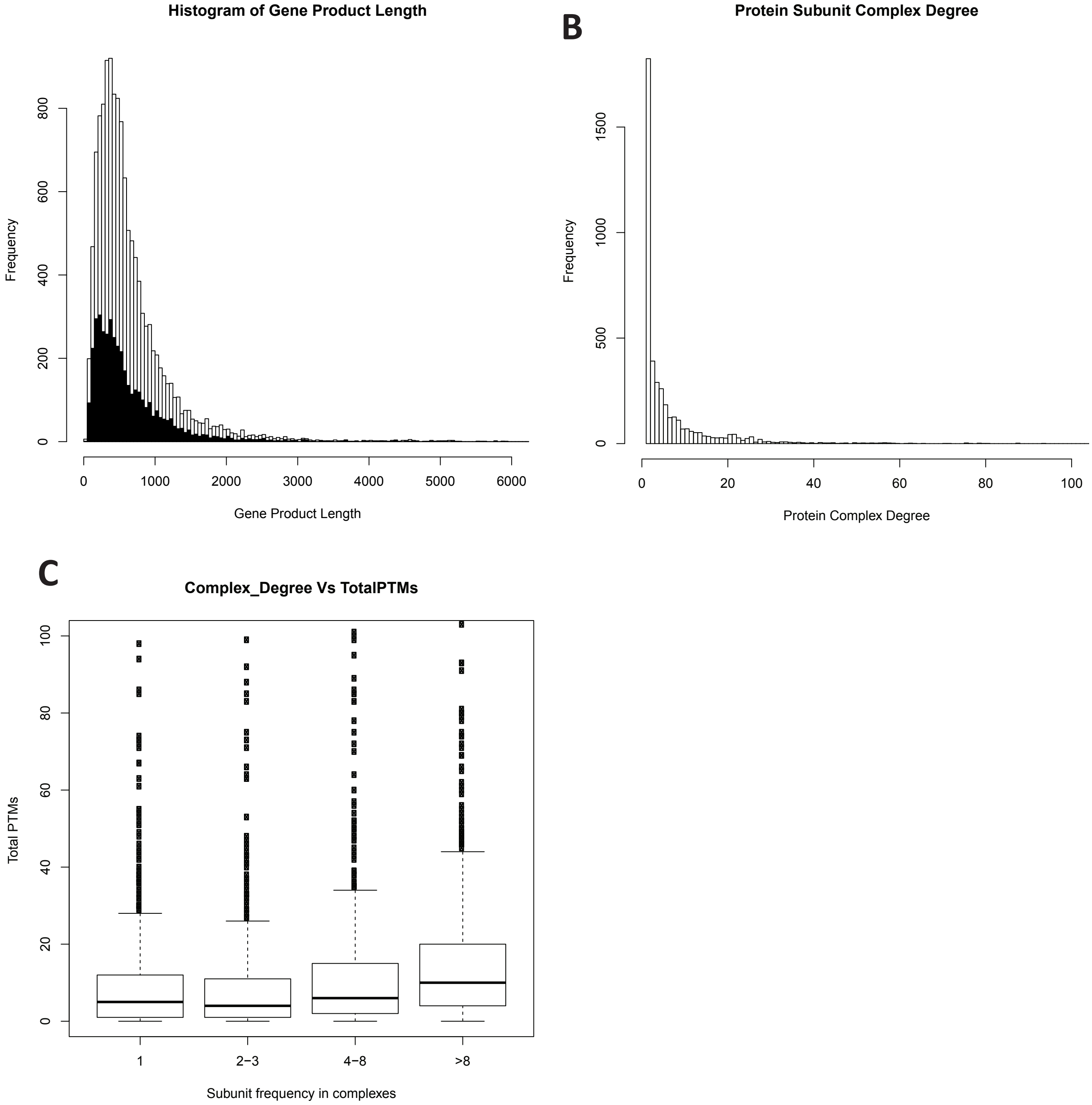
Histogram of Gene Product Length
Frequency
0
200
400
600
800
0
1000
2000
3000
4000
5000
6000
Gene Product Length
B
Protein Subunit Complex Degree
Frequency
0
500
1000
1500
0
20
40
60
80
100
Protein Complex Degree
C
Complex_Degree Vs TotalPTMs
Total PTMs
0
20
40
60
80
100
1
2−3
4−8
>8
Subunit frequency in complexes
